# Magnetohydrodynamics and viscosity variation in couple stress squeeze film lubrication between rough flat and curved circular plates

**DOI:** 10.1038/s41598-023-50326-7

**Published:** 2023-12-27

**Authors:** Haewon Byeon, Y. L. Latha, B. N. Hanumagowda, Vediyappan Govindan, A. Salma, Sherzod Abdullaev, Jagadish. V. Tawade, Fuad A. Awwad, Emad A. A. Ismail

**Affiliations:** 1 Department of AI Big Data, Inje University, Gimhae, 50834 South Korea; 2 Department of Mathematics, School of Applied Sciences, REVA University, Bangalore, 560064 India; 3 Department of Mathematics, Hindustan Institute of Technology and Science, Rajiv Gandhi Salai (OMR), Padur, Kelambakkam, Tamil Nadu 603103 India; 4 Faculty of Chemical Engineering, New Uzbekistan University, Tashkent, Uzbekistan; 5 Department of Science and Innovation, Tashkent State Pedagogical University Named After Nizami, Bunyodkor Street 27, Tashkent, Uzbekistan; 6 Department of Mathematics, Vishwakarma University, Pune, 411048 India; 7 Department of Quantitative Analysis, College of Business Administration, King Saud University, P.O. Box 71115, 11587 Riyadh, Saudi Arabia; 8 Department of Quantitative Analysis, College of Business Administration, King Saud University, P.O. Box 71115, 11587 Riyadh, Saudi Arabia

**Keywords:** Engineering, Mathematics and computing, Nanoscience and technology

## Abstract

A simplified mathematical model has been developed for understanding combined effects of surface roughness, viscosity variation and couple stresses on the squeeze film behaviour of a flat and a curved circular plate in the presence of transverse magnetic field. The Stokes (1966) couple stress fluid model is included to account for the couple stresses arising due to the presence of microstructure additives in the lubricant. In the context of Christensen’s (1969) stochastic theory for the lubrication of rough surfaces, two types of one-dimensional roughness patterns (radial and azimuthal) are considered. The governing modified stochastic Reynolds type equations are derived for these roughness patterns. Expressions for the mean squeeze film characteristics are obtained. Numerical computations of the results show that the azimuthal roughness pattern on the curved circular and flat plate results in more pressure buildup whereas performance of the squeeze film suffers due to the radial roughness pattern. Further the Lorentz force characterized by the Hartmann number, couple stress parameter and viscosity variation parameter improve the performance of the squeeze film lubrication as compared to the classical case (Non-magnetic, Newtonian case and non-viscous case).

## Introduction

When two lubricated surfaces are moving towards one another at normal speed, the squeeze film lubrication phenomena occur. A cushion is created by the tiny layer of lubricant that lies between the two surfaces, preventing quick contact between them. The squeeze film lubrication phenomenon is observed in several applications such as gears, bearings, machine tools, rolling elements and automatic engines, dampers, and human joints. Several researchers^1,2^ examined the squeeze film bearings using Newtonian lubricants. The usage of fluids combined with various additives has drawn a lot of interest. A micro continuum theory of couple stress fluids has been presented by Stokes^3^ to explain the unique flow behaviour of non-Newtonian fluids. This micro continuum theory depicts the rotational field in terms of velocity field and allows for polar effects including the presence of couple stresses, body couples, and nonsymmetric stress tensors. The couple stress fluid model is important for engineering and scientific applications of pumping fluids such as synthetic lubricants, colloidal fluids, biofluids, and liquid crystals. The couple stress fluid theory was adopted by several tribology researchers to examine various bearing systems. Ramanaiah^4^ and Lin^5,6^ to investigate the significant problems of hydrodynamic lubrication.

Magnetohydrodynamic (MHD) is the study of dynamics of flow of electrically conducting fluid with the application of magnetic field. The MHD bearings with conducting fluids possess high thermal conductivity and high electrical conductivity features compared to conventional bearings. The magnetic field is an important factor in conditioning and controlling tribological property. The effect of a magnetic field on the non-Newtonian fluid has great importance in engineering applications; for instance, MHD generators, accelerators, and purification of crude oil. MHD also finds applications in physiological process such as magnetic therapy. Since magnetic field can enhance a bearing's load capacity, several theoretical investigations are carried out such as, Shukla^7^, Lin^8^ and Kumza^9^ have analyzed the influence of magnetic fields on different squeeze film bearings in a wide range of circumstances. Recent research by Naduvinamani et al.^10^ and Hanumagowda et al.^11,12^ on the combined effects of MHD and couple stress squeeze film for various models revealed that the presence of non-Newtonian fluid enhances the characteristics of the squeeze film when comparing to the Newtonian and non-magnetic cases.

While viscosity varies with both pressure and temperature, in the above literature viscosity assumed it to be constant. Since viscosity is a function of temperature and pressure, the change in viscosity with temperature must be considered in many practical situations as the lubricant has to function under an extensive range of temperatures. Thus, many kinds of research are taking place related to viscosity variation due to temperature and pressure^13^. Several researchers have examined the impact of couple-stress fluid and variations in viscosity. For instance, Lin et al.^14,15^ investigated the effect of non-Newtonian fluid and pressure-dependent viscosity on the squeeze film lubrication of circular and wide parallel plates. Their findings suggest that the impact of viscosity variation leads to an increase in load and prolongs the squeeze film time. The combined influence of PDV and couple stress on the lubrication of the squeeze film has been demonstrated by Hanumagowda^16^ for circular step plates, that result to an enhancement of the characteristics of the squeeze film, which is comparable to what is seen in the iso-viscosity lubricant conditions.

In all the above-mentioned studies it has been observed that the work is limited only for the smooth surfaces. But practically, even the rough surface is very crucial. In engineering science and real-world scenarios, the impact of surface roughness is significant. In bearings, surface roughness is a measure of the texture of a surface. It is quantified by the vertical deviations of a real surface from its ideal form. If these deviations are large, the surface is rough; if they are small, the surface is smooth. Rough surfaces typically have higher friction coefficients and wear faster than smooth ones. Since deviations in the surface may serve as the starting point for cracks or corrosion, roughness is frequently a reliable indicator of how well a mechanical component will operate. In many cases, the roughness asperity heights are of the same order as the mean separation between the lubricated contacts. Now, it has been well established that, the surface roughness of the bearing surfaces significantly affects the bearing performance especially in the boundary or mixed lubrication. To study rough surfaces, Christensen^17^ proposed a stochastic model. The impact of surface roughness on the characteristics of squeeze film in the presence of couple stress fluids has been examined by numerous researchers, including Prakash and Tiwari^18^, Gupta et al.^19^, and Lin^20,21^. These scholars have explored different geometries and have reached comparable conclusions regarding the influence of roughness on bearings. All these studies have considered the viscosity of the fluid with the assumption that it is constant, ignoring any effects that pressure might have on the substance. Lin et al.^22^ for long partial journal bearings, Kumar and Sachidanand^23^ for short journal bearings, and Siddangouda et al.^24^ for long journal bearings report on the influence of viscosity variation and surface roughness.

Numerous studies^25–32^ in recent years investigated at the effects of PVD with couple stress fluid on rough plates, few are Naduvinamani et al.^33^ for parallel circular plate, Ayyappa et al.^34^ for Short Journal Bearings, Hanumagowda et al.^35^ for stepped circular plate and Noor Jahan et al.^36^ for annular plate. All these researchers looked into how roughness influence lubrication characteristics when MHD and couple stress was present, and they all came to the same conclusion: that pressure, load carrying capacity, and the approach of the squeeze film time were all decreased (or increased) on a bearing surface with a radial (or an azimuthal) roughness pattern when compared with the smooth case. References^32,37–40^ reports some recent development versus fluid flow with various flow assumptions. Li et al.^41^, Kong et al.^42^ and Shi et al.^43^ recently explored the characteristics of surface pressure pulsation of centrifugal pump magnetic liquid, micro-spectroscopy under high pressure and thermal and pressure coupled effects respectively. Bai et al.^44^ examined the thermo-mechanical mechanism subject to soil particle re-arrangement of thermodynamics granular. Bian et al.^45^, Zhu et al.^46^ and Zhang et al.^47^ respectively worked bioinspired magnetism-responsive hybrid microstructures, generalized micro-fluid rectifiers via anisotropic slippery hollow tracks and nonlinear hysteretic behaviours with strain-stiffening of magnetorheological gel composite applications. Attributes of cavity dynamics, trajectories and forces on vertical water entries, shear thickening fluids and behaviour between fluid and structure from coupling system respectively investigated by Lyu et al.^48^, Sun et al.^49^ and Huang et al.^50^. Employed in the reverse roll coating process, non-isothermal couple stress fluid was used by Hiremath^51^ and Shahzad^52^ to investigate the effects of a magnetic field on a curved circular plate and a flat plate lubricated with non-Newtonian fluid and slide effects.

In view of these results and the resulting effects, the authors of the current study projected that MHD, viscosity variation, and surface roughness each perform an impact in the couple stress squeeze film lubrication between flat plate and a curved circular plate. Pressure, load bearing capacity, and squeeze film time are examined as a function of a wide range of physical parameters. The results reveal numerous interesting behaviours which require more study of the equations that describe non-Newtonian couple stress fluid phenomena with variable viscosity.

### Mathematical formulation of the problem

**Figure 1:**
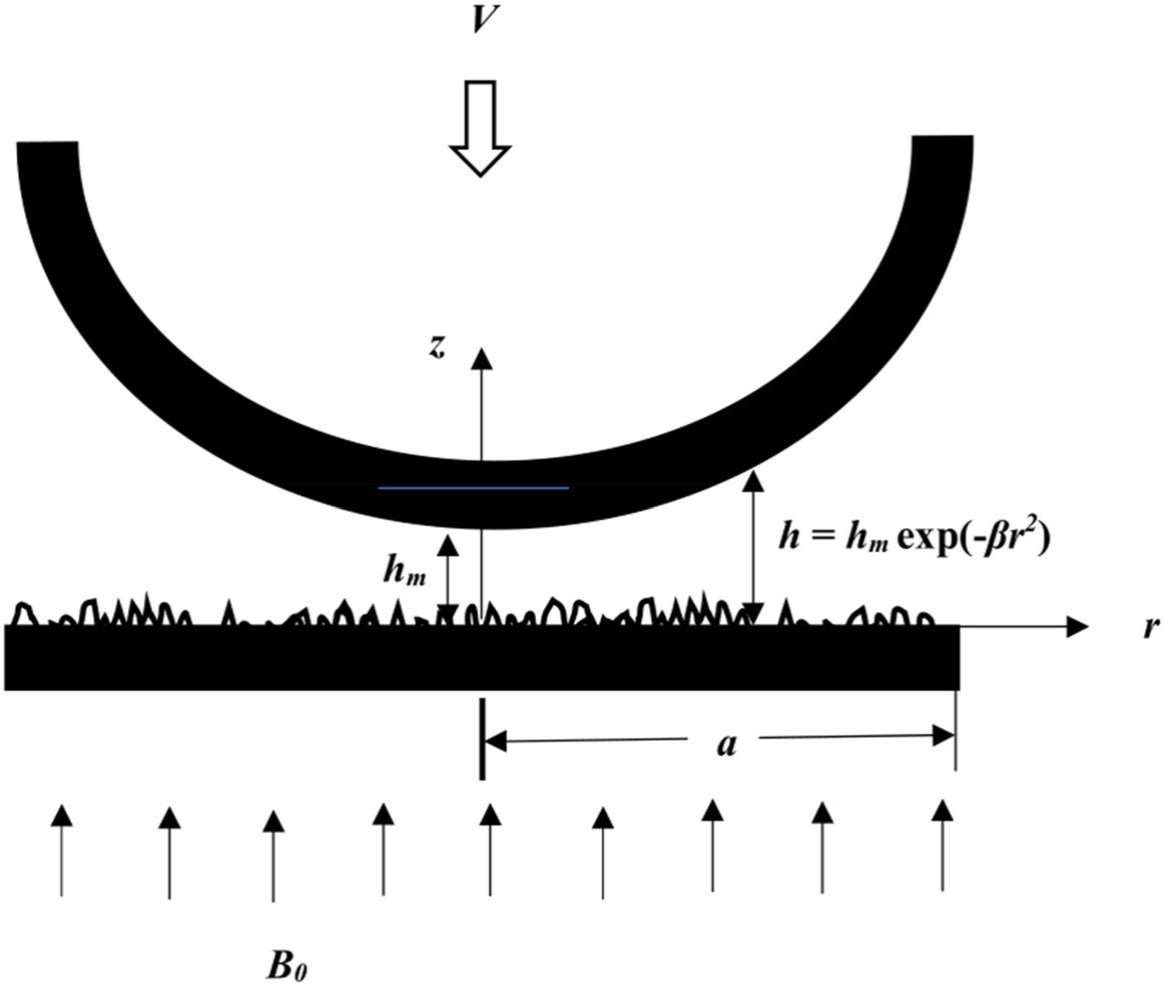
Geometry of curved circular and rough flat plates.

Consider the squeeze film mechanism between a curved circular and flat plate as shown in Fig. 1. Under a constant load, the upper plate is moving at a squeezing velocity *V* towards the lower rough flat plate. The lubricant in the system is taken to be an isothermal, incompressible electrically conducting fluid. A uniform transverse magnetic field B_0_ is applied in the z-direction. Assume that the fluid film is thin, the fluid inertia is small, the body force is negligible except for the Lorentz force, and the induced magnetic field is small compared to the applied field. The continuity equation and governing equation of motion of the lubricant which are discussed by Lin et al.^53^ are given as:

1 $$\frac{{\partial^{2} u}}{{\partial z^{2} }} - \frac{\eta }{\mu }\frac{{\partial^{4} u}}{{\partial z^{4} }} - \frac{\sigma }{\mu }B_{0}^{2} u = \frac{1}{\mu }\frac{\partial p}{{\partial r}}$$

2 $$\frac{\partial p}{{\partial z}} = 0$$

3 $$r\frac{\partial w}{{\partial z}} + \frac{\partial }{\partial r}(ru) = 0$$

where $u$ and $w$ are the components of velocities in $r$ and $z$ direction respectively, $p$ represents the pressure in the region, $\eta$ is material constant responsible for couple stress, $\sigma$ is the electrical conductivity, $\mu$ is lubricant viscosity and $B_{0}$ is the applied magnetic field.

The relevant boundary conditions are as follows:

On the surface of the upper curved plate ($z = h$)

4a $$u = 0\,\,\left( {{\text{No}} - {\text{Slip}}} \right),\,\,\,\frac{{\partial^{2} u}}{{\partial z^{2} }} = 0\,\,\left( {{\text{Vanishing}}\,\,{\text{of}}\,\,{\text{couple}}\,\,{\text{stress}}} \right)$$

4b $$w = V\,\,\left( {{\text{Squeezing}}\,\,{\text{Velocity}}} \right)$$

On the surface of the lower flat plate ($z = 0$)

5a $$u = 0\,\,\left( {\text{No - Slip}} \right),\,\,\frac{{\partial^{2} u}}{{\partial z^{2} }} = 0\,\,\left( {{\text{Vanishing}}\,\,{\text{of}}\,\,{\text{couple}}\,\,{\text{stress}}} \right)$$

5b $$w = 0$$

The height of the film has been designated by

6 $$h = h_{m} \,\exp \left( { - \beta r^{2} } \right),\,\,\,\,0\,\, \le \,\,r\,\, \le a$$

where $h_{m}$ is the minimum film thickness.

### Solution method

Using no-slip and vanishing of couple stresses on the solid boundary, the solution of Eq. (1) is

8 $$u = \left\{ {\left( {\Delta_{1} - \Delta_{2} } \right) - 1} \right\}\frac{{h_{0}^{2} }}{{\mu M_{0}^{2} }}\frac{\partial p}{{\partial r}}$$

9a $${\text{Here}},\,\,\Delta_{1} = \lambda_{11} ,\,\,\,\Delta_{2} = {\kern 1pt} \lambda_{12} \,\,\,{\text{for}}{\kern 1pt} {\kern 1pt} {\kern 1pt} {{\,\,\,\,4M_{0}^{2} l^{2} } \mathord{\left/ {\vphantom {{\,\,\,\,4M_{0}^{2} l^{2} } {h_{0}^{2} }}} \right. \kern-0pt} {h_{0}^{2} }} < 1$$

9b $$\Delta_{1} = \lambda_{21} ,\,\,\,{\kern 1pt} \Delta_{2} = {\kern 1pt} \lambda_{22} \,\,\,{\kern 1pt} {\text{for}}{{\,\,\,\,4M_{0}^{2} l^{2} } \mathord{\left/ {\vphantom {{\,\,\,\,4M_{0}^{2} l^{2} } {h_{0}^{2} }}} \right. \kern-0pt} {h_{0}^{2} }} = 1$$

9c $$\Delta_{1} = \lambda_{31} ,\,\,\Delta_{2} \, = \,\,\lambda_{32} {\kern 1pt} \,\,{\text{for}}{\kern 1pt} {{\,\,\,\,4M_{0}^{2} l^{2} } \mathord{\left/ {\vphantom {{\,\,\,\,4M_{0}^{2} l^{2} } {h_{0}^{2} }}} \right. \kern-0pt} {h_{0}^{2} }} > 1$$

The associated relations in Eqs. (9a), (9b) and (9c) are given in Appendix.

Substituting Eqn., (8) in continuity Eqn., (3) and integrating subject to the boundary conditions (4b) & (5b) we get the modified Reynolds equation, as

10 $$\frac{\partial }{\partial r}\left\{ {\frac{1}{\mu }r \, \xi (h,l,M_{0} )\frac{\partial p}{{\partial r}}} \right\} = - rV{\kern 1pt}$$

where,

$$\xi (h,l,M_{0} ) = \left\{ {\begin{array}{*{20}l} {\frac{{h_{0}^{2} }}{{M_{0}^{2} }}\left\{ {\frac{2l}{{(\lambda_{1}^{2} - \lambda_{2}^{2} )}}\left( {\frac{{\lambda_{2}^{2} }}{{\lambda_{1} }}\tanh \frac{{\lambda_{1} h}}{2l} - \frac{{\lambda_{1}^{2} }}{{\lambda_{2} }}\tanh \frac{{\lambda_{2} h}}{2l}} \right) + h{\kern 1pt} } \right\}} \hfill & {{\text{for}}\,\,\,{{4M_{0}^{2} l^{2} } \mathord{\left/ {\vphantom {{4M_{0}^{2} l^{2} } {h_{0}^{2} < }}} \right. \kern-0pt} {h_{0}^{2} < }}1} \hfill \\ {\frac{{h_{0}^{2} }}{{M_{0}^{2} }}\left\{ {\frac{h}{2}Sech^{2} \left( {\frac{h}{2\sqrt 2 l}} \right) - 3\sqrt 2 l\tan h\left( {\frac{h}{2\sqrt 2 l}} \right) + h} \right\}} \hfill & {{\text{for}}\,\,\,{\kern 1pt} {\kern 1pt} {{4M_{0}^{2} l^{2} } \mathord{\left/ {\vphantom {{4M_{0}^{2} l^{2} } {h_{0}^{2} = 1}}} \right. \kern-0pt} {h_{0}^{2} = 1}}} \hfill \\ {\frac{{h_{0}^{2} }}{{M_{0}^{2} }}\left\{ {\frac{2l}{{M_{0} }}\left\{ {\frac{{\left( {A_{2} Cot\theta - B_{2} } \right)SinB_{2} h - \left( {B_{2} Cot\theta + A_{2} } \right)SinhA_{2} h}}{{CosB_{2} h + CoshA_{2} h}}} \right\} + h} \right\}} \hfill & {{\text{for}}\,\,\,{\kern 1pt} {{4M_{0}^{2} l^{2} } \mathord{\left/ {\vphantom {{4M_{0}^{2} l^{2} } {h_{0}^{2} > }}} \right. \kern-0pt} {h_{0}^{2} > }}1} \hfill \\ \end{array} } \right.$$

The pressure dependent viscosity derived by Barus et al.^54,55^ is

11 $$\mu = \mu_{0} e^{\alpha p}$$

where $\alpha$ denotes the coefficient of viscosity and $\mu_{0}$ is the viscosity at ambient pressure and at a constant temperature.

In view of the discussions regarding stochastic model for roughness carried out by Christensen^17^ the film thickness is assumed to be

$$H = \,h + h_{f} (r,\theta ,\kappa )$$

wherein $h$ denotes the smooth and unstressed part of the film thickness and $h_{f}$ is the part due to surface roughness measured from the mean level and its random character is expressed by the variable $\kappa$ . $h_{f}$ is governed by the probability density function

$$\varsigma (h_{f} ) = \left\{ {\begin{array}{*{20}l} {\frac{35}{{32n^{7} }}(n^{2} - h_{f}^{2} )^{3} ,{\kern 1pt} } \hfill & { - n < h_{f} < n} \hfill \\ 0 \hfill & {{\text{elsewhere}}} \hfill \\ \end{array} } \right.$$

where $n = 3\overline{\sigma }$ and $\overline{\sigma }$ is standard deviation.

Taking the stochastic average of modified Reynolds Eq. (10) with respect to $\varsigma (h_{f} )$, the stochastic modified Reynolds equation is obtained as

12 $$\frac{1}{r}\frac{\partial }{\partial r}\left\{ {\frac{{e^{ - \alpha E(p)} }}{{\mu_{0} }}E\left\{ {\xi (H,l,M_{0} )} \right\}r\frac{\partial E(p)}{{\partial r}}} \right\} = - V$$

where, $$E\left( \cdot \right) = \int\limits_{ - \infty }^{\infty } {\left( \cdot \right)\varsigma (h_{f} )dh_{f} }$$.

With respect to Christensen’s theory^17^ for roughness, there are two kinds of one-dimensional roughness configuration such as radial and azimuthal roughness configuration.

### Radial Roughness

One-dimensional pattern of radial roughness appears like a star pattern made up of long, thin ridges and valleys travelling in the radial direction, and, in this case, the film thickness is $H = h + h_{f} (\theta ,\kappa )$,

The average modified Reynolds Eq. (12) takes form,

13 $$\frac{1}{r}\frac{\partial }{\partial r}\left\{ {\frac{{e^{ - \alpha E(p)} }}{{\mu_{0} }}E\left\{ {\xi (H,l,M_{0} )} \right\}r\frac{\partial E(p)}{{\partial r}}} \right\} = - V$$

### Azimuthal Roughness

For a film thickness of, the azimuthal roughness pattern presents as an array of long, thin ridges and valleys running in the $\theta$- direction.

$$H = h + h_{f} (r,\kappa )$$,

and the averaged modified Reynolds Eq. (12) takes the form

14 $$\frac{1}{r}\frac{\partial }{\partial r}\left\{ {\frac{{e^{ - \alpha E(p)} }}{{\mu_{0} }}\left\{ {E\left( {\frac{1}{{\xi (H,l,M_{0} )}}} \right)} \right\}^{ - 1} r\frac{\partial E(p)}{{\partial r}}} \right\} = - V$$

Equations (13) and (14) collectively written as

15 $$\frac{1}{r}\frac{\partial }{\partial r}\left\{ {\frac{{e^{ - \alpha E(p)} }}{{\mu_{0} }}S\left( {H,l,M_{0} ,n} \right)r\frac{\partial E(p)}{{\partial r}}} \right\} = - V$$

where,$$S(H,\,l,\,M_{0} ,\,n) = \left\{ {\begin{array}{*{20}l} {E(\xi (H,l,M_{0} ))} \hfill & {{\text{for}}\,\,{\text{radial}}\,\,{\text{roughness}}} \hfill \\ {\left\{ {E\left( {\frac{1}{{\xi (H,l,M_{0} )}}} \right)} \right\}^{ - 1} } \hfill & {{\text{for}}{\kern 1pt} \,\,{\text{azimuthal}}\,\,{\text{roughness}}} \hfill \\ \end{array} } \right.$$

$$E(\xi (H,l,M_{0} )) = \frac{35}{{32n^{7} }}\int\limits_{ - n}^{n} {\xi (H,l,M_{0} )(n^{2} - h_{f}^{2} )^{3} dh_{f} }$$,

$$E\left( {\frac{1}{{\xi (H,l,M_{0} )}}} \right) = \frac{35}{{32n^{7} }}\int\limits_{ - n}^{n} {\frac{{(n^{2} - h_{f}^{2} )^{3} }}{{\xi (H.l,M_{0} )}}dh_{f} }$$.

In Eq. (15) incorporates the subsequent non-dimensional quantities.

$$r^{*} = \frac{r}{a},\,\,C = \frac{n}{{h_{0} }},\,\,h_{m}^{*} = \frac{{h_{m} }}{{h_{0} }},\,\,h^{*} = \frac{h}{{h_{0} }},\,\,l^{*} = \frac{2l}{{h_{0} }},\,\,P^{*} = - \frac{{h_{0}^{3} p}}{{\mu_{0} a^{2} V}},\,\,G = - \frac{{\alpha \mu_{0} a^{2} V}}{{h_{0}^{3} }},$$

$$h^{*} = h_{m}^{*} \exp ( - \beta r^{*2} )$$, $$\beta = \gamma a^{2}$$.

For film pressure, the Reynolds equation is

16 $$\frac{1}{{r^{*} }}\frac{d}{{dr^{*} }}\left\{ {e^{{ - GP^{*} }} S^{*} \left( {H^{*} ,l^{*} ,M_{0} ,C} \right)r^{*} \frac{{dP^{*} }}{{dr^{*} }}} \right\} = - 1$$

where,$$S^{*} (H^{*} ,l^{*} ,M_{0} ,C) = \left\{ {\begin{array}{*{20}l} {E(\xi^{*} (H^{*} ,l^{*} ,M_{0} )),} \hfill & {{\text{for}}{\kern 1pt} {\kern 1pt} {\kern 1pt} {\text{radial}}{\kern 1pt} {\kern 1pt} \,{\text{roughness}}} \hfill \\ {\left\{ {E\left( {\frac{1}{{\xi^{*} (H^{*} ,l^{*} ,M_{0} )}}} \right)} \right\}^{ - 1} ,} \hfill & {{\text{for}}{\kern 1pt} {\kern 1pt} \,{\text{azimuthal}}{\kern 1pt} {\kern 1pt} \,{\text{roughness}}} \hfill \\ \end{array} } \right.$$

$$E(\xi^{*} (H^{*} ,l^{*} ,M_{0} )) = \frac{35}{{32C^{7} }}\int\limits_{ - C}^{C} {\xi^{*} (H^{*} ,l^{*} ,M_{0} )(C^{2} - h_{f}^{2} )^{3} dh_{f} }$$,

$$E\left( {\frac{1}{{\xi^{*} (H^{*} ,l^{*} ,M_{0} )}}} \right) = \frac{35}{{32n^{7} }}\int\limits_{ - C}^{C} {\frac{{(C^{2} - h_{f}^{2} )^{3} }}{{\xi^{*} (H^{*} ,l^{*} ,M_{0} )}}dh_{f} }$$

$$\xi^{*} (H^{ * } ,l^{ * } ,M_{0} ) = \left\{ \begin{gathered} \frac{1}{{M_{0}^{2} }}\left\{ {\frac{{l^{ * } }}{{(\lambda_{1}^{*2} - \lambda_{2}^{*2} )}}\left( {\frac{{\lambda_{2}^{*2} }}{{\lambda_{1}^{*} }}\tanh \left( {\frac{{\lambda_{1}^{*} H^{ * } }}{{l^{ * } }}} \right) - \frac{{\lambda_{1}^{*2} }}{{\lambda_{2}^{*} }}\tanh \left( {\frac{{\lambda_{2}^{*} H^{ * } }}{{l^{ * } }}} \right)} \right) + H^{ * } } \right\}{\kern 1pt} {\kern 1pt} {\kern 1pt} {\kern 1pt} {\kern 1pt} {\kern 1pt} {\kern 1pt} {\kern 1pt} {\kern 1pt} {\kern 1pt} {\kern 1pt} {\kern 1pt} {\kern 1pt} {\kern 1pt} {\kern 1pt} {\kern 1pt} {\kern 1pt} {\kern 1pt} {\kern 1pt} {\kern 1pt} \,\,\,\,\,\,\,\,\,\,\,\,\,\,\,\,\,\,\,{\text{for}}{\kern 1pt} {\kern 1pt} {\kern 1pt} M_{0}^{2} l^{*2} < 1 \hfill \\ \frac{1}{{M_{0}^{2} }}\left\{ {\frac{{H^{*} }}{2}Sech^{2} \left( {\frac{{H^{*} }}{{\sqrt 2 l^{*} }}} \right) - \frac{{3l^{*} {\kern 1pt} }}{\sqrt 2 }{\kern 1pt} {\kern 1pt} tanh\left( {\frac{{H^{*} }}{{\sqrt 2 l^{*} }}} \right) + H^{*} } \right\}{\kern 1pt} {\kern 1pt} {\kern 1pt} {\kern 1pt} {\kern 1pt} {\kern 1pt} {\kern 1pt} {\kern 1pt} {\kern 1pt} {\kern 1pt} {\kern 1pt} {\kern 1pt} {\kern 1pt} {\kern 1pt} {\kern 1pt} {\kern 1pt} {\kern 1pt} {\kern 1pt} {\kern 1pt} {\kern 1pt} {\kern 1pt} {\kern 1pt} {\kern 1pt} {\kern 1pt} {\kern 1pt} {\kern 1pt} {\kern 1pt} {\kern 1pt} {\kern 1pt} {\kern 1pt} {\kern 1pt} {\kern 1pt} {\kern 1pt} {\kern 1pt} {\kern 1pt} {\kern 1pt} {\kern 1pt} {\kern 1pt} {\kern 1pt} {\kern 1pt} {\kern 1pt} {\kern 1pt} {\kern 1pt} {\kern 1pt} {\kern 1pt} {\kern 1pt} {\kern 1pt} {\kern 1pt} {\kern 1pt} {\kern 1pt} {\kern 1pt} {\kern 1pt} {\kern 1pt} {\kern 1pt} {\kern 1pt} {\kern 1pt} {\kern 1pt} {\kern 1pt} {\kern 1pt} {\kern 1pt} {\kern 1pt} {\kern 1pt} {\kern 1pt} {\kern 1pt} {\kern 1pt} {\kern 1pt} {\kern 1pt} {\kern 1pt} {\kern 1pt} {\kern 1pt} {\kern 1pt} {\kern 1pt} {\kern 1pt} {\kern 1pt} {\kern 1pt} {\kern 1pt} {\kern 1pt} {\kern 1pt} {\kern 1pt} {\kern 1pt} {\kern 1pt} {\kern 1pt} {\kern 1pt} {\kern 1pt} {\kern 1pt} {\kern 1pt} {\kern 1pt} {\kern 1pt} {\kern 1pt} {\kern 1pt} {\kern 1pt} {\kern 1pt} {\kern 1pt} {\kern 1pt} {\kern 1pt} {\kern 1pt} {\kern 1pt} {\kern 1pt} {\kern 1pt} {\kern 1pt} {\kern 1pt} {\kern 1pt} {\kern 1pt} {\kern 1pt} {\kern 1pt} \,\,\,\,\,\,\,\,\,\,\,{\text{for}}\,\,M_{0}^{2} l^{*2} = 1 \hfill \\ \frac{1}{{M_{0}^{2} }}\left\{ {\frac{{l^{*} \left( {A_{2}^{*} Cot\theta^{*} - B_{2}^{*} } \right)Sin\left( {B_{2}^{*} H^{*} } \right) - l^{*} \left( {B_{2}^{*} Cot\theta^{*} + A_{2}^{*} } \right)Sinh\left( {A_{2}^{*} H^{*} } \right)}}{{M_{0} \left( {Cos\left( {B_{2}^{*} H^{*} } \right) + Cosh\left( {A_{2}^{*} H^{*} } \right)} \right)}} + H^{*} {\kern 1pt} } \right\}{\text{ for}}{\kern 1pt} {\kern 1pt} {\kern 1pt} {\kern 1pt} M_{0}^{2} l^{*2} > 1 \hfill \\ \end{gathered} \right.$$

$$\lambda_{1}^{*} = \sqrt {\frac{{1 + \sqrt {\left( {1 - M_{0}^{2} l^{ * 2} } \right)} }}{2}} \,\,\,\,\lambda_{2}^{*} = \sqrt {\frac{{1 - \sqrt {\left( {1 - M_{0}^{2} l^{ * 2} } \right)} }}{2}}$$

$$A_{2}^{*} = \sqrt {\frac{{2M_{0} }}{{l^{*} }}} Cos\left( {\frac{{\theta^{*} }}{2}} \right)\,\,\,{\kern 1pt} {\kern 1pt} B_{2}^{*} = \sqrt {\frac{{2M_{0} }}{{l^{*} }}} Sin\left( {\frac{{\theta^{*} }}{2}} \right),\,\,\theta^{*} = \tan^{ - 1} \left( {\sqrt {l^{*2} M_{0}^{2} - 1} } \right){\kern 1pt}$$

The pressure boundary conditions are

17 $$\frac{{dP^{*} }}{{dr^{*} }}\,\, = \,\,0\,\,{\text{at}}\,\,r^{*} \, = \,0,\,\,\,{\text{and}}\,\,\,P^{*} \, = \,0\,\,{\text{at}}\,\,r^{*} \, = 1$$

Integrating the Eq. (16) with respect to r^*^ and using the pressure boundary conditions (17), we get dimensionless pressure as

18 $$P^{*} = - \frac{1}{G}\ln \left\{ {1 + \int\limits_{1}^{{r^{*} }} {\frac{{Gr^{*} }}{{2S^{*} \left( {H^{*} ,l^{*} ,M_{0} ,C} \right)}}dr^{*} } } \right\}$$

The load-carrying capacity in dimensionless form:

19 $$W^{*} = \frac{{Wh_{0}^{3} }}{{\mu_{0} a^{3} ({{dh} \mathord{\left/ {\vphantom {{dh} {dt}}} \right. \kern-0pt} {dt}})}} = - \frac{1}{G}\int\limits_{0}^{1} {\left[ {\ln \left\{ {1 + \int\limits_{1}^{{r^{*} }} {\frac{{Gr^{*} }}{{2S^{*} \left( {H^{*} ,l^{*} ,M_{0} ,C} \right)}}dr^{*} } } \right\}} \right]r^{*} dr^{*} }$$

The dimensionless form of the squeeze film time relation is

20 $$T^{*} = \frac{{th_{0}^{2} }}{{\mu_{0} a^{3} }} = - \frac{1}{G}\int\limits_{{h_{1}^{*} }}^{1} {\left[ {\int\limits_{0}^{1} {\ln \left\{ {1 + \int\limits_{1}^{{r^{*} }} {\frac{{Gr^{*} }}{{2S^{*} \left( {H^{*} ,l^{*} ,M_{0} ,C} \right)}}dr^{*} } } \right\}r^{*} dr^{*} } } \right]} dh_{m}^{*}$$

The shear stress along the surface is given by.

The components of stress tensor required for calculating frictional force is

21 $$\tau_{zr} = \mu \left. {\frac{\partial u}{{\partial z}}} \right|_{z = 0} - \eta \left. {\frac{{\partial^{3} u}}{{\partial z^{3} }}} \right|_{z = 0}$$

The frictional force at $z = 0$ is given by

$$F = \int\limits_{0}^{1} {\left( {t_{zr} } \right)_{z = 0} dr}$$

$$F = \frac{1}{{\sigma B_{0}^{2} }}\frac{AB}{{(A^{2} - B^{2} )l}}\int\limits_{0}^{1} {\frac{\partial p}{{\partial r}}\left[ {B^{3} \tan h\frac{Ah}{{2l}} - A^{3} tanh\frac{Bh}{{2l}}} \right]dr}$$

The non-dimensional frictional force is given by

22 $$F^{*} = - \frac{{Fh_{0} }}{{\mu_{0} a^{2} V}} = \frac{1}{{M_{0}^{2} }}\frac{{2A^{*} B^{*} }}{{(A^{*2} - B^{*2} )l^{*} }}\int\limits_{0}^{1} {\frac{{dP^{*} }}{{dr^{*} }}\left[ {B^{3} \tan h\frac{{A^{*} h^{*} }}{{l^{*} }} - A^{*3} tanh\frac{{B^{*} h^{*} }}{{l^{*} }}} \right]dr^{*} }$$

where $$\frac{{dP^{*} }}{{dr^{*} }} = - \frac{{r^{*} e^{{GP^{*} }} }}{{2G^{*} \left( {H^{*} ,l^{*} ,M_{0} ,C} \right)}}$$.

## Results and discussion

Based on Stoke’s^3^ theory for couple stress fluid lubrication and Christensen’s^17^ theory for surface roughness, the impact of MHD, couple stress, and viscosity variation on squeeze film lubrication between rough flat and curved circular plate is examined in the current analysis. The squeeze film characteristics are analyzed for various non-dimensional parameters such as the Hartmann number $M_{0}$, couple stress parameter $l^{*}$, roughness parameter *C* and viscosity variation parameter *G*. The following range of parameters is considered in this study: *M*_*0*_ = 0 to 5, l^*^ = 0 to 0.4, *C* = 0 to 0.4, *G* = 0 to 0.008.

### Limiting cases

1. As *M*_*0*_ → 0, l^*^ → 0, *G* → 0, the present analysis reduces to the results obtained by Naduvinamani and Gurubasavaraj^56^.
2. As *G* → 0, the present analysis reduces to the results obtained by Lin et al.^53^.

#### Squeeze-film pressure

**Figure 2:**
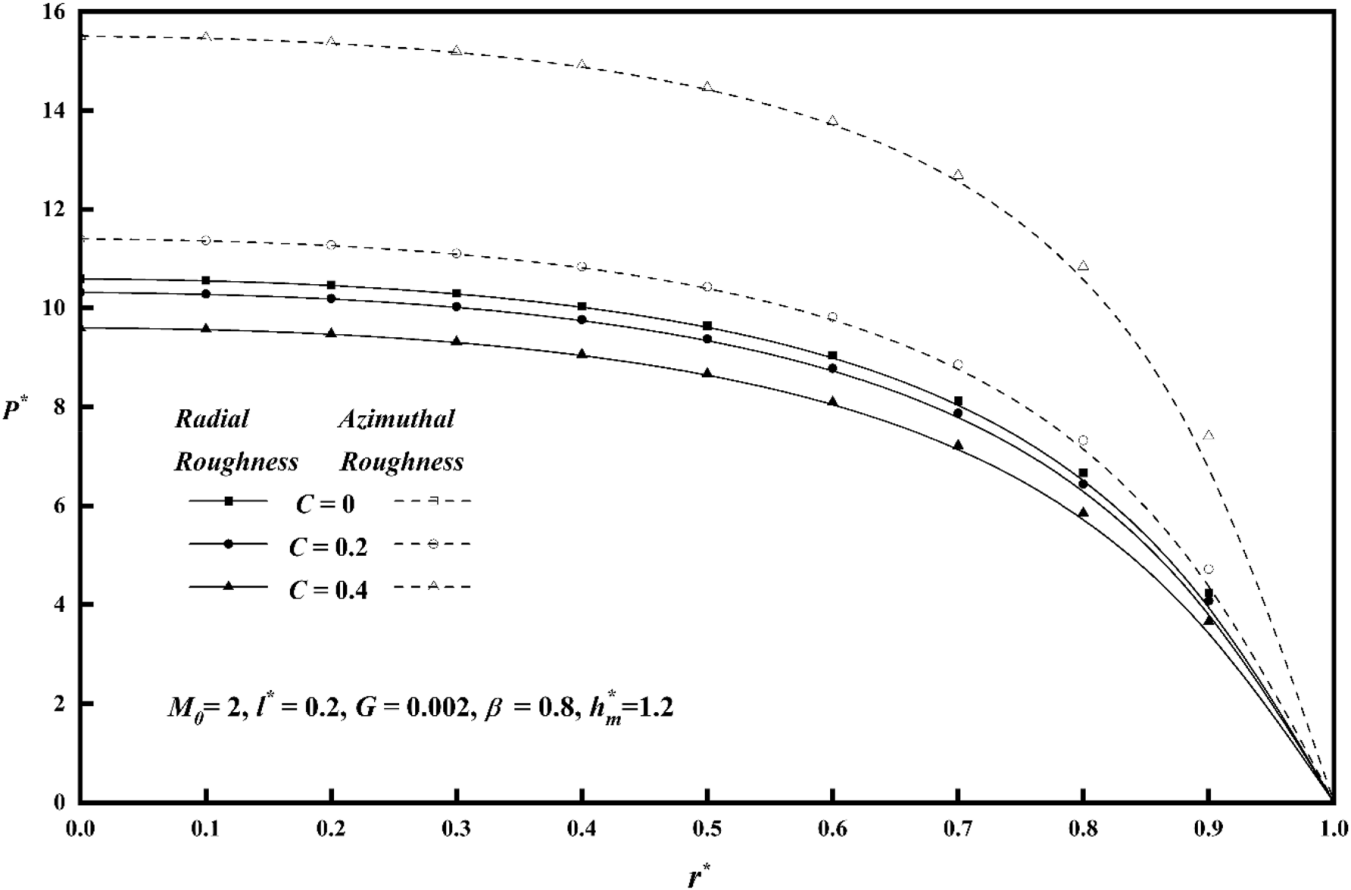
Comparison of $P^{*}$ with $r^{*}$ on a graph for various values of *C.*

Figure 2 shows, for both roughness patterns, how the pressures $P^{*}$ and $r^{*}$ vary as a function of the roughness parameter *C* when other parameters are held fixed*.* This results the pressure $P^{*}$ value of the azimuthal roughness to increases while the radial roughness decreases. As the vertical deviations of the surface increase, correspondingly rises the squeeze-film pressure.

**Figure 3:**
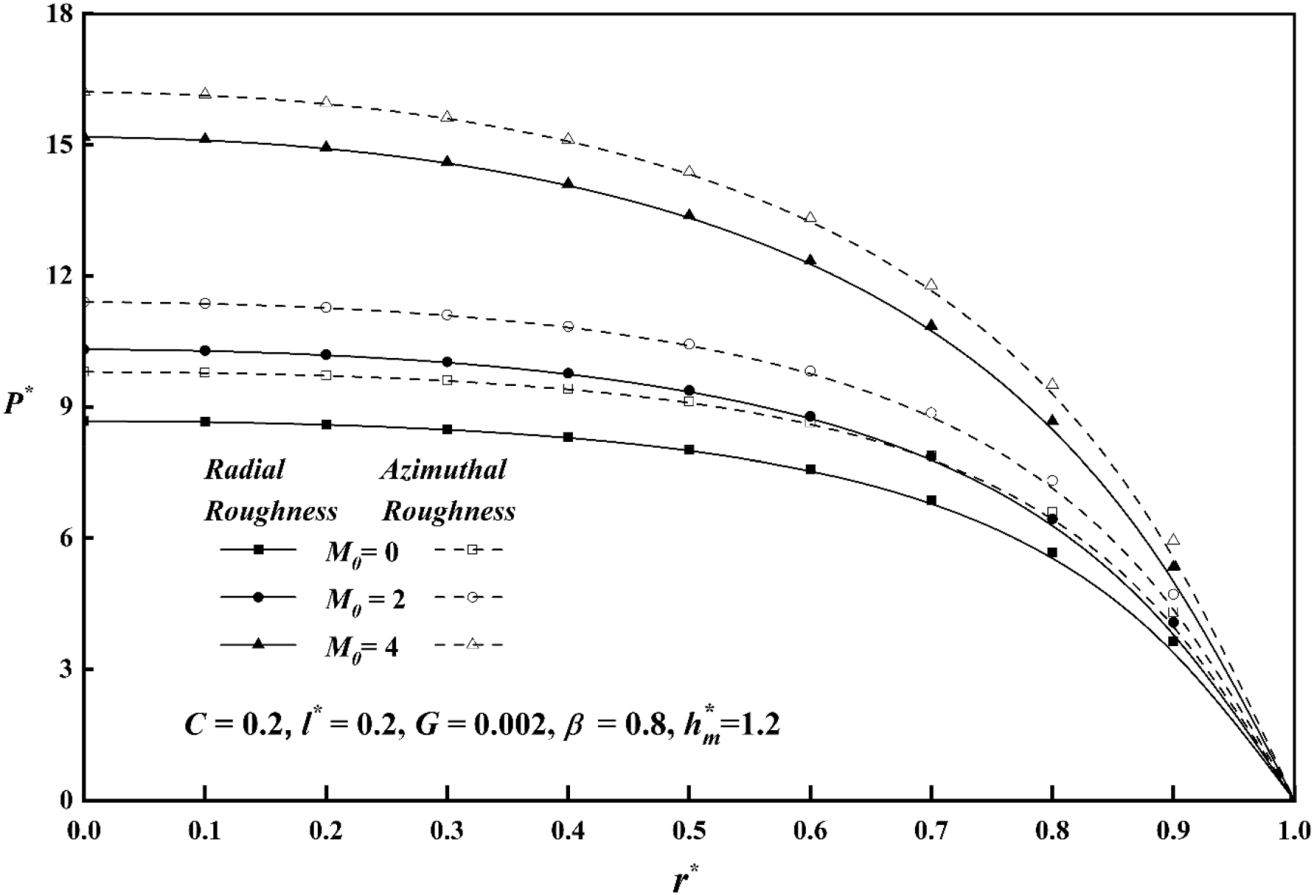
Comparison of $P^{*}$ with $r^{*}$ on a graph for various values of $M_{0}$.

**Figure 4:**
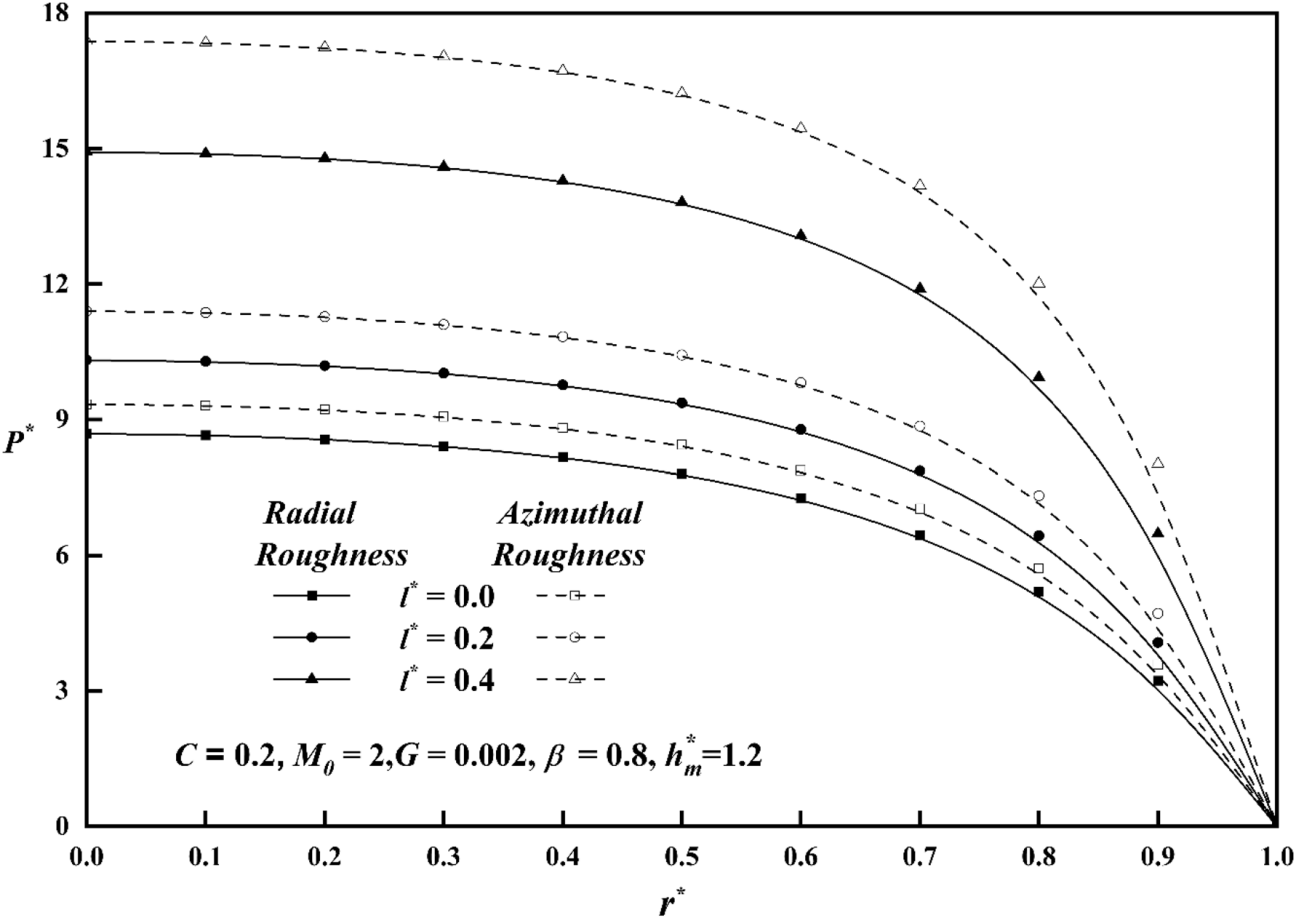
Comparison of $P^{*}$ with $r^{*}$ on a graph for various values of $l^{*}$.

**Figure 5:**
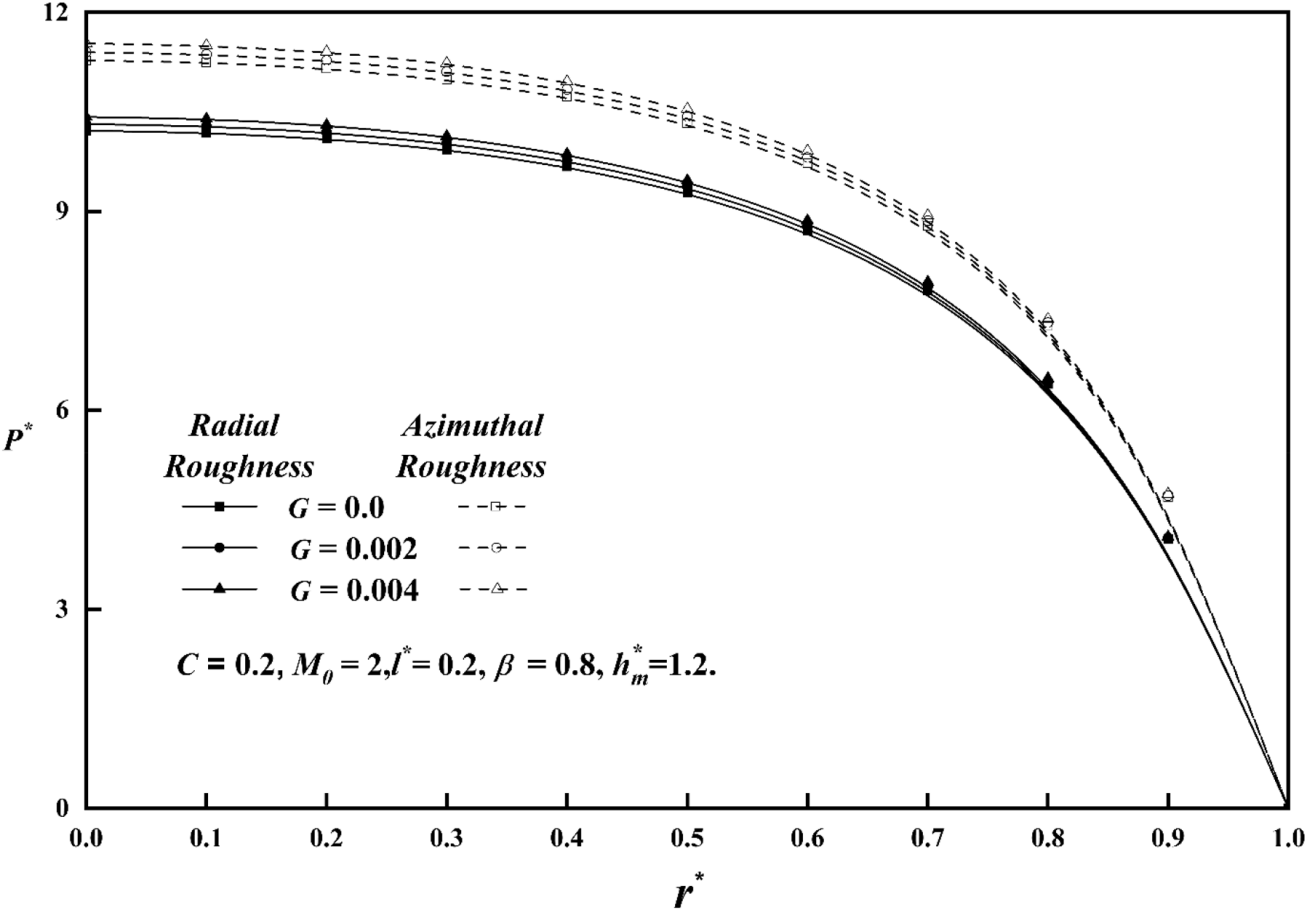
Comparison of $P^{*}$ with $r^{*}$ on a graph for various values of $G$.

Figure 3 displays the pressure in the fluid film region as a function of the Hartmann number for both roughness patterns and in comparison, to a nonmagnetic case $\left( {M_{0} = { 0}} \right)$, it is seen that the pressure distribution rises as $M_{0}$ increases. As Hartmann number values rise, the lubricant becomes more magnetised, which in turn interacts with the applied magnetic field. Additionally, a magnetic field that is provided perpendicular to the flow decreases the fluid's velocity in the film region, retaining a significant volume of fluid and the pressure distribution is produced by this fluid. Figure 4 illustrates the pressure $P^{*}$ versus $r^{*}$ variance for different values of the couple stress parameter l^*^ for both roughness patterns, and noticed that the $P^{*}$ rises for increasing $l^{*}$ values. Because the couple-stress fluid provides higher resistance to the flowing fluid, a greater amount of fluid remains in the region which generates large pressure distribution. It is also seen that $P^{*}$ is more emphasized for the azimuthal than the radial roughness configuration. Figure 5 explains the change of $P^{*}$ against $r^{*}$ for distinct values of viscosity parameter $G$ for both roughness configurations and noticed that the squeeze-film pressure improves considerably with increasing value of $G$. This is because increase in viscosity parameter results in increased lubricating film strength, which reduces the possibility of direct contact between the contacting surfaces. This reduces frictional wear and damage, resulting in improved system durability and efficiency.

**Figure 6:**
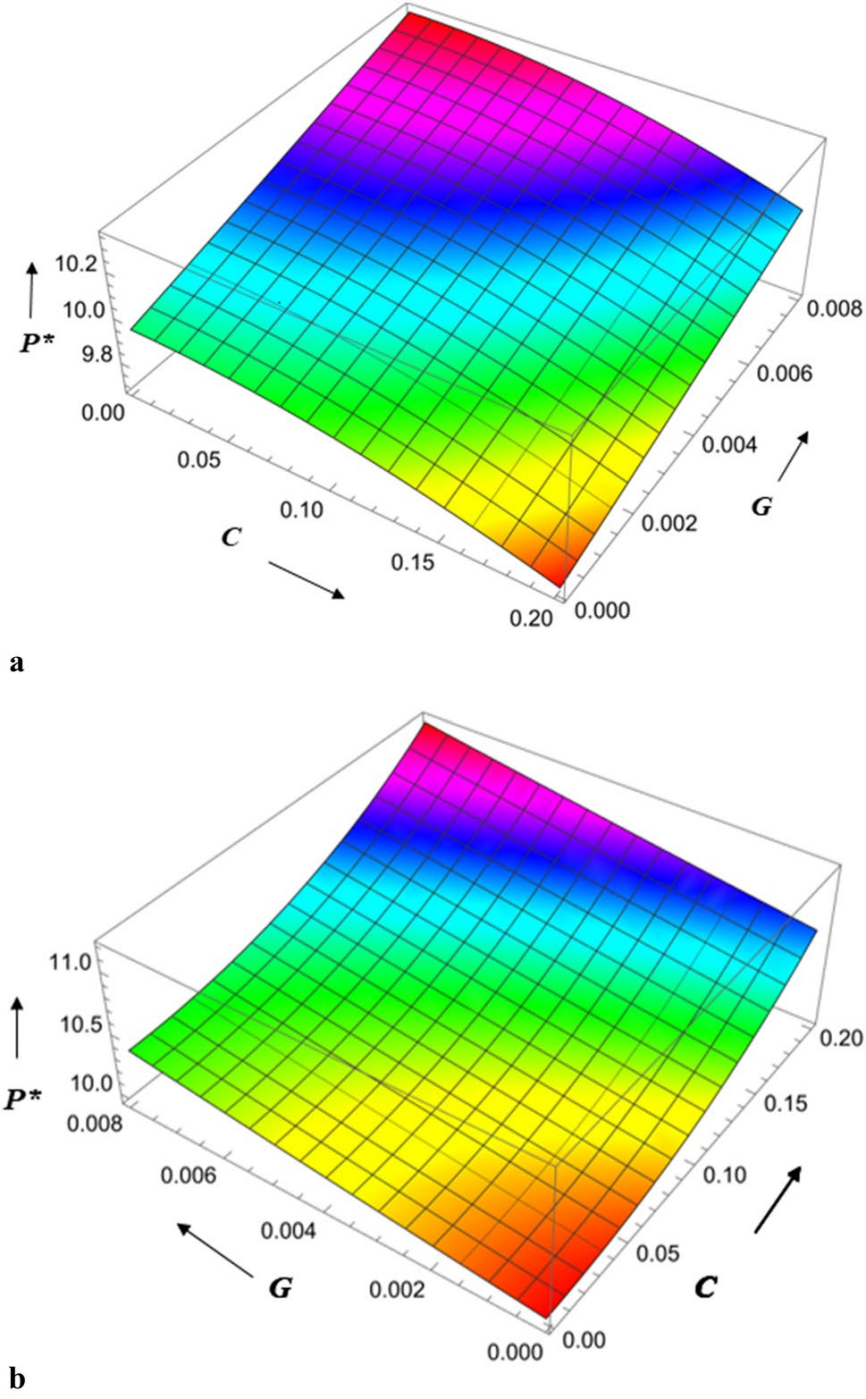
(**a**) Diagram showing how *C* (radial roughness) and *G* influences $P^{*}$. (**b**) Diagram showing how *C* (azimuthal roughness) and *G* influences $P^{*}$.

**Figure 7:**
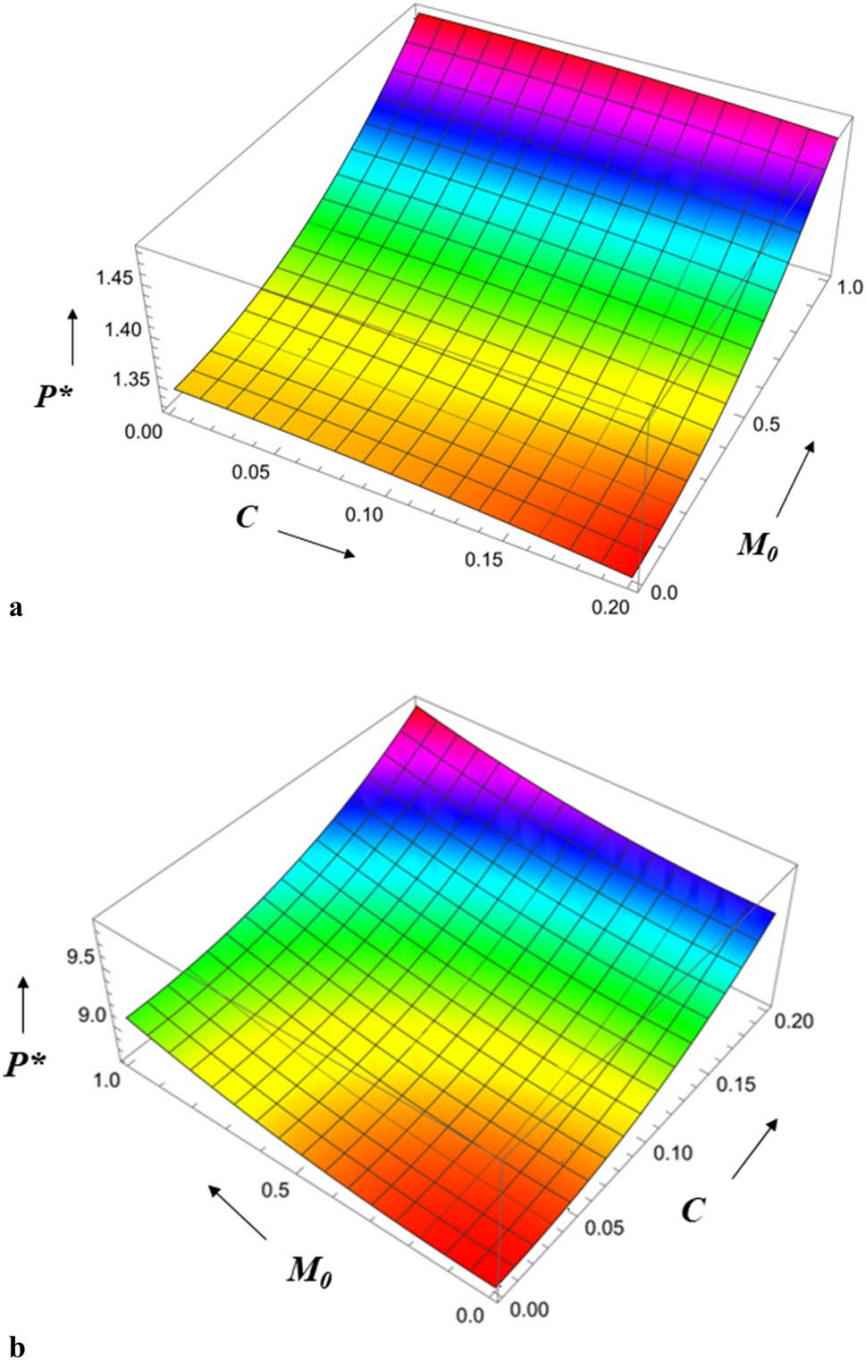
(**a**) Diagram showing how *C* (radial roughness) and $M_{0}$ influences $P^{*}$. (**b**) Diagram showing how *C* (azimuthal roughness) and $M_{0}$ influences $P^{*}$.

The combined effect of surface roughness and viscosity variation on pressure is presented in Fig. 6a,b. It is observed that the radial roughness parameter is minimum, the viscosity variation is greater, and the pressure is maximum. Also, the azimuthal roughness parameter is higher, the viscosity variation is higher, and the pressure is higher. It is noted that an increase in the viscosity parameter increases pressure more for azimuthal than radial roughness parameters. The combined effect of the roughness parameter and magnetic parameter is presented in Fig. 7a,b. It is observed that pressure is maximum when the radial roughness parameter is less and the Hartmann number is higher, and when the azimuthal roughness is higher, and the Hartmann number is higher. It is noted that an increase in Hartmann number increases pressure for azimuthal roughness more than radial roughness. As a result, the Hartmann number, roughness, and viscosity parameter are all beneficial for improving pressure distribution.

#### Load carrying capacity

**Figure 8:**
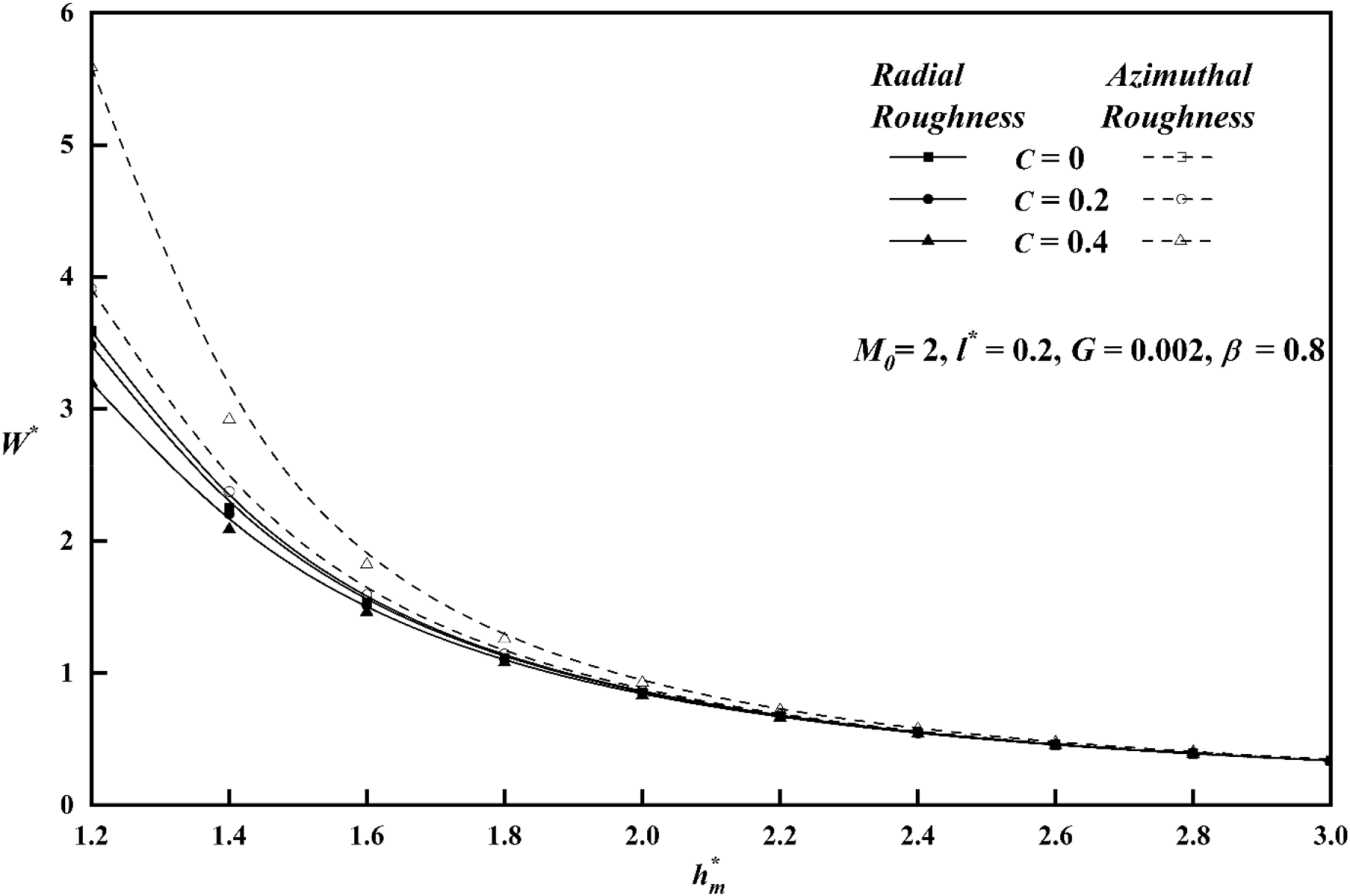
Plot of $W^{*}$ with $h_{m}^{*}$ for varying values of *C.*

**Figure 9:**
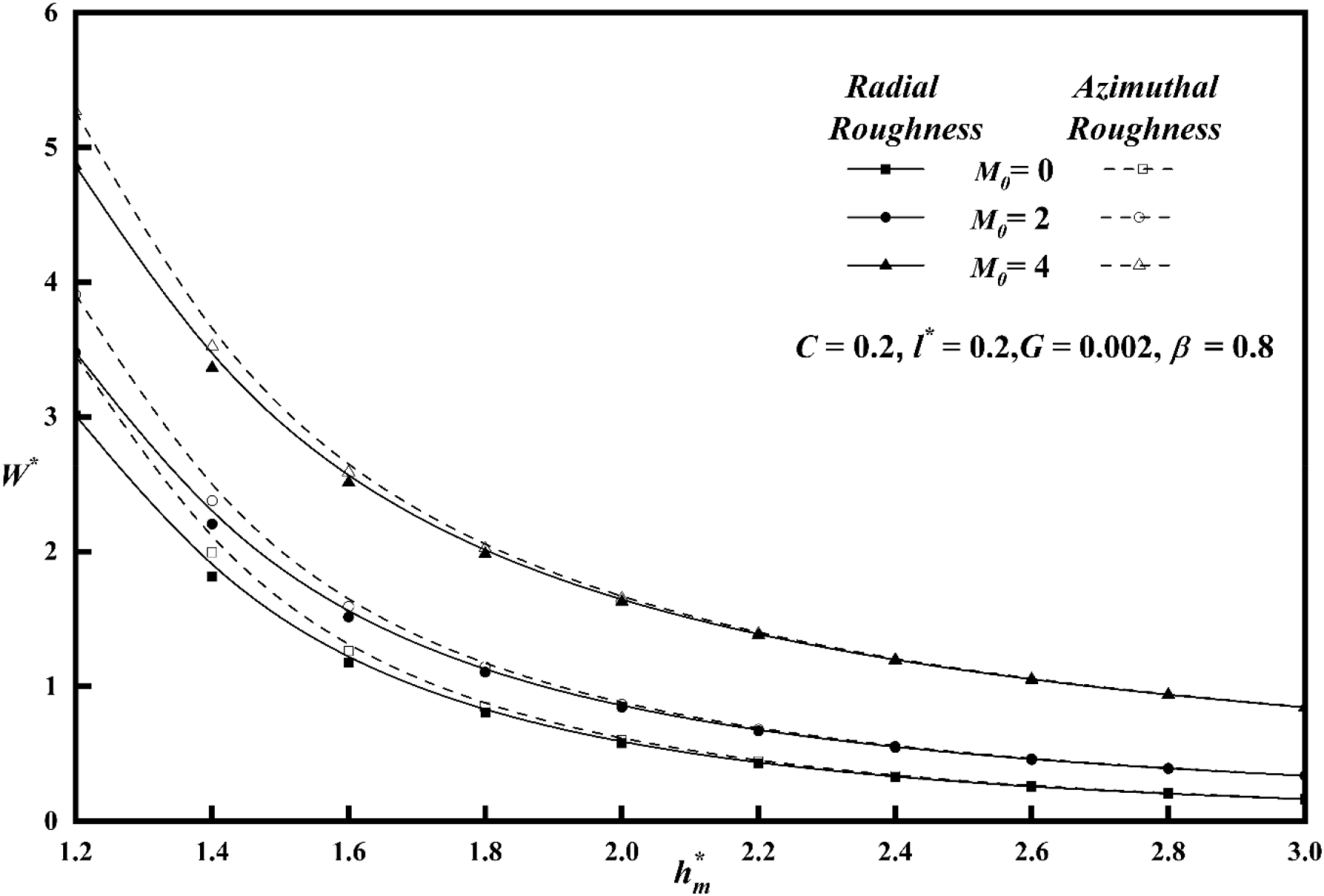
Plot of $W^{*}$ with $h_{m}^{*}$ for varying values of $M_{0}$.

**Figure 10:**
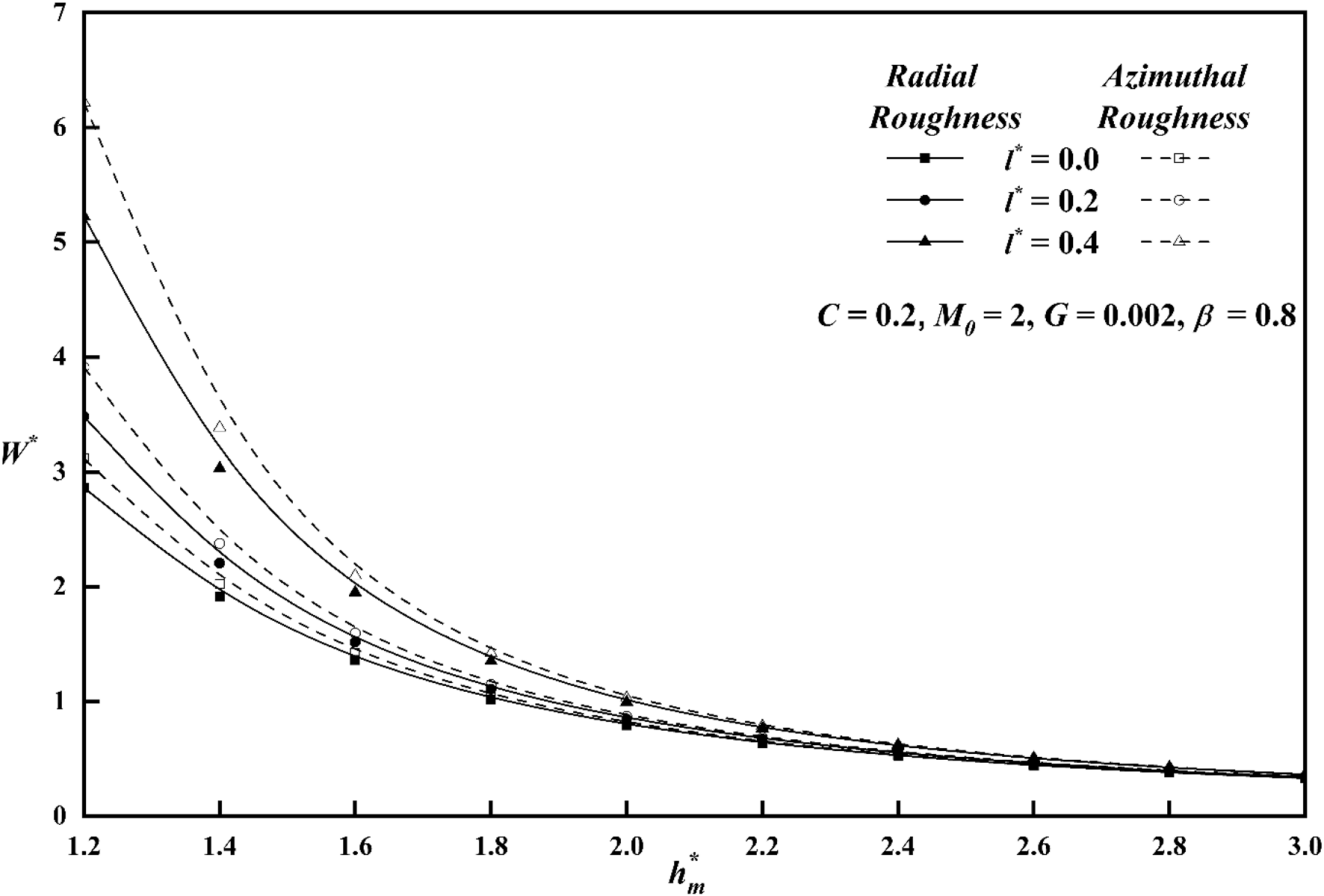
Plot of $W^{*}$ with $h_{m}^{*}$ for varying values of $l^{*}$.

**Figure 11:**
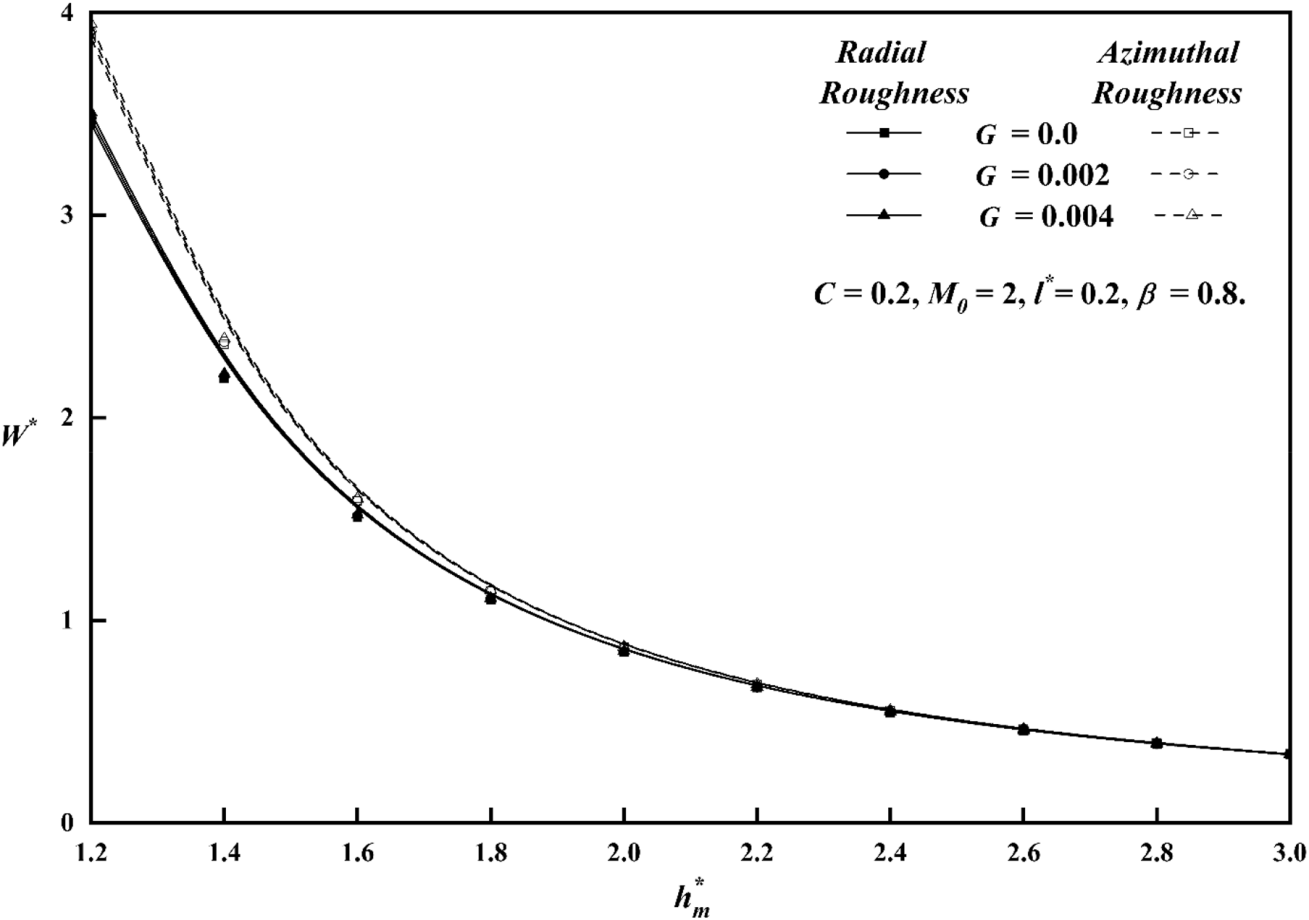
Plot of $W^{*}$ with $h_{m}^{*}$ for varying values of $G$.

To comprehend the impacts of various physical quantities, it is essential to graphically represent the relationship between load-carrying capacity and other relevant variables. Figure 8 depicts the relationship between the roughness parameter *C* and the load carrying capacity $W^{*}$ along $h_{m}^{*}$ keeping other parameters constant for both roughness patterns. The presence of azimuthal/radial roughness patterns has been seen to result in an increase/decrease in the value of $W^{*}$ when compared to the smooth case $\left( {i.e\,\,\,C = 0} \right)$. At a value of $C = 0.4$, which corresponds to large vertical deviations, it is seen that the change in $W^{*}$ is more noticeable for the azimuthal roughness pattern compared to the radial roughness pattern. The azimuthal roughness pattern exhibits a significantly higher load compared to the radial roughness pattern. The deviation of $W^{*}$ versus $h_{m}^{*}$ for distinct $M_{0}$ values is elaborated in Fig. 9 for both the roughness configurations and from the figure it is found that as contrasted to magnetic case (i.e. $M_{0} = 0$), $W^{*}$ increases for increasing values $M_{0}$. A graph of load $W^{*}$ against $h_{m}^{*}$ is depicted in Fig. 10, showcasing different values of l^*^ for both roughness configurations. When compared to Newtonian case, the impact of the coupe stress parameter enhances the load along the azimuthal roughness while decreasing it along the radial roughness. According to Fig. 11, which shows the change of $W^{*}$ versus $h_{m}^{*}$ as a function of $G$ for both roughness patterns we note that the load-carrying capacity of a lubrication system, can be significantly enhanced when the viscosity parameter of the lubricant increases. The main reason for this improvement is the lubricant's capacity to provide a thicker and more durable lubricating film between the moving surfaces. Also, from all figures, show that as $h_{m}^{*}$ increases consequently does the load's inverse.

**Figure 12:**
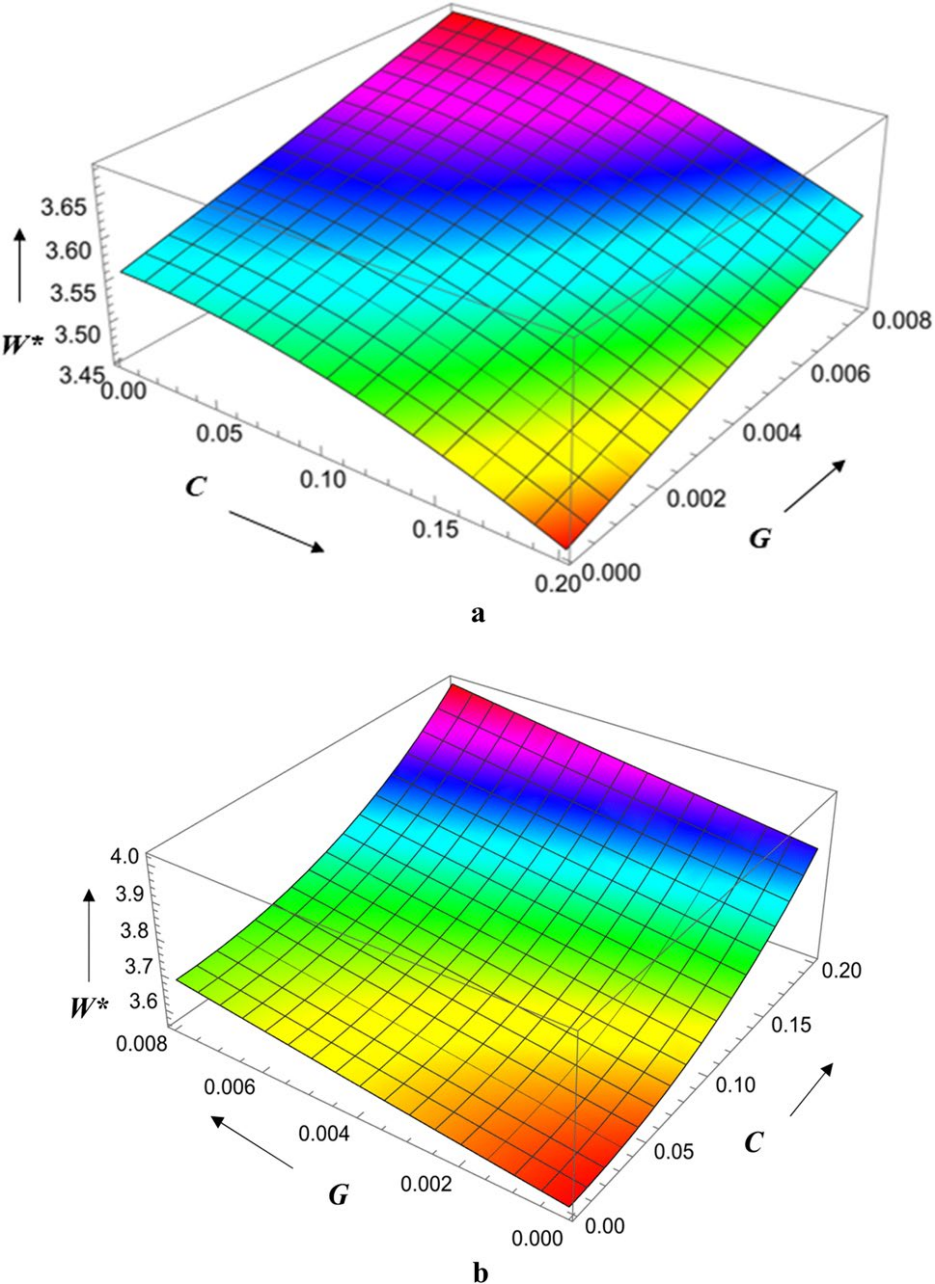
(**a**) Diagram showing how *C* (radial roughness) and *G* influences $W^{*}$. (**b**) Diagram showing how *C* (azimuthal roughness) and *G* influences $W^{*}$.

**Figure 13:**
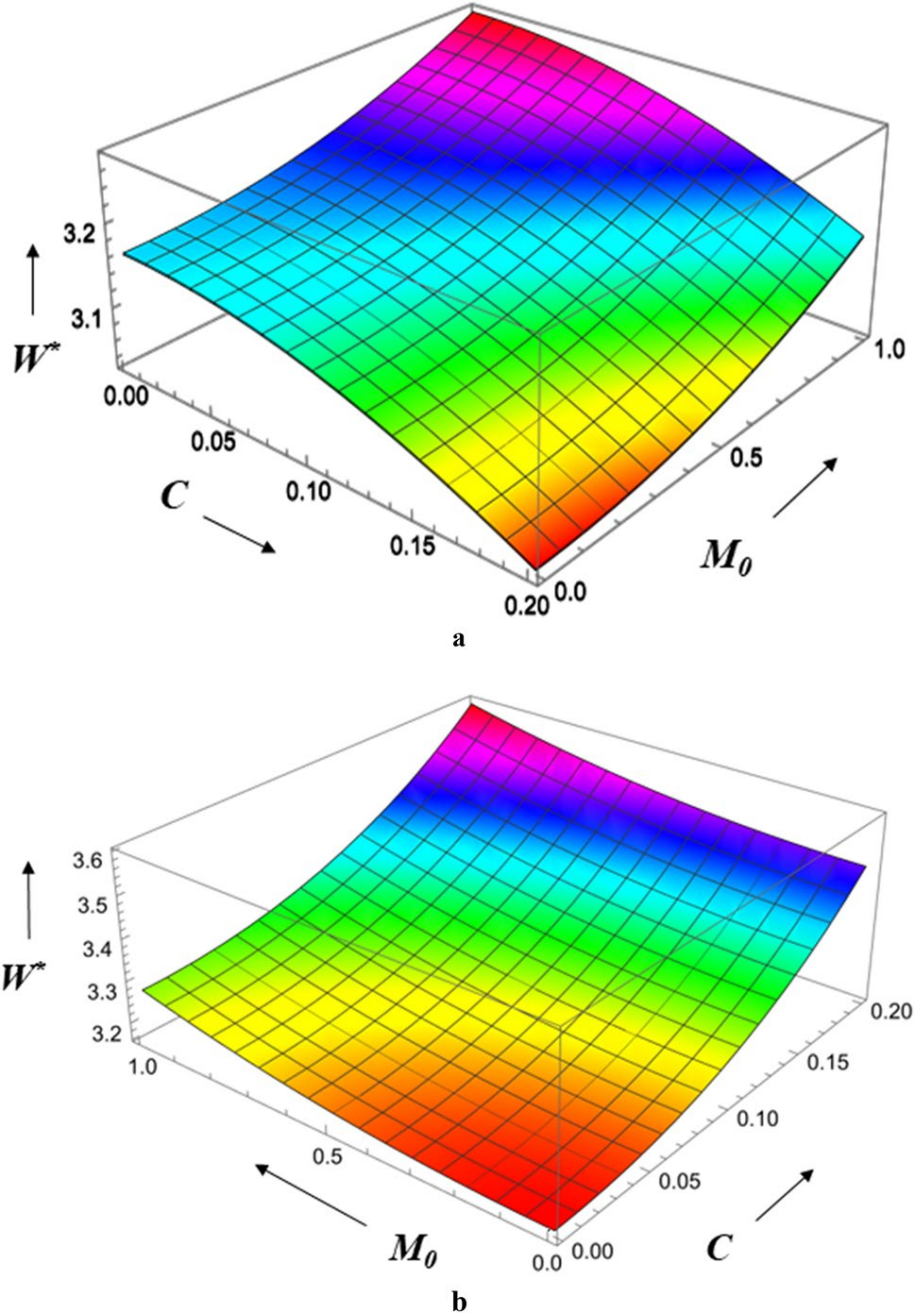
(**a**) Diagram showing how *C* (radial roughness) and $M_{0}$ influences $W^{*}$. (**b**) Diagram showing how *C* (azimuthal roughness) and $M_{0}$ influences $W^{*}$.

Figure 12a,b show the combined effect of surface roughness and viscosity variation on load-carrying capacity. The magnetic parameter is larger, the load bearing capacity is greatest, and the radial roughness parameter is the smallest it can be. The result is an increase in magnetic parameter strength, load bearing capacity, and azimuthal roughness parameter. The Hartmann number has a greater effect on load bearing capacity for azimuthal roughness than radial roughness. Figure 13a,b show the combined effect of the roughness parameter and the magnetic parameter on load-carrying capacity. We observe that the highest load bearing capacity occurs at small values of the radial roughness parameter and large values of the Hartmann number, and that the same holds true for the azimuthal direction. The Hartmann number has a bigger impact on load-carrying capacity for azimuthal roughness than radial roughness.

#### Squeeze film time

**Figure 14:**
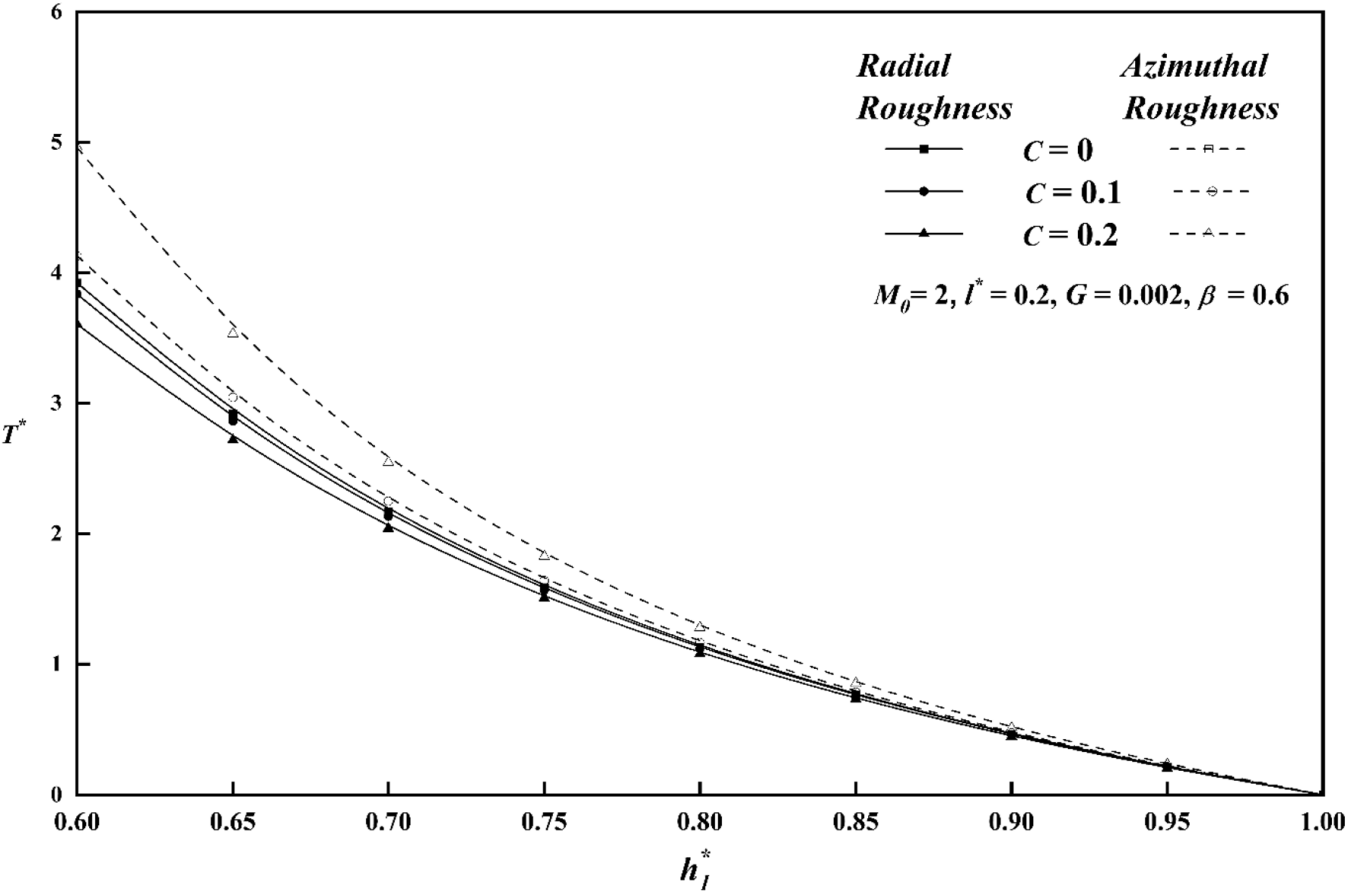
Comparison of $T^{*}$ with $h_{1}^{*}$ on a graph for various values of *C.*

**Figure 15:**
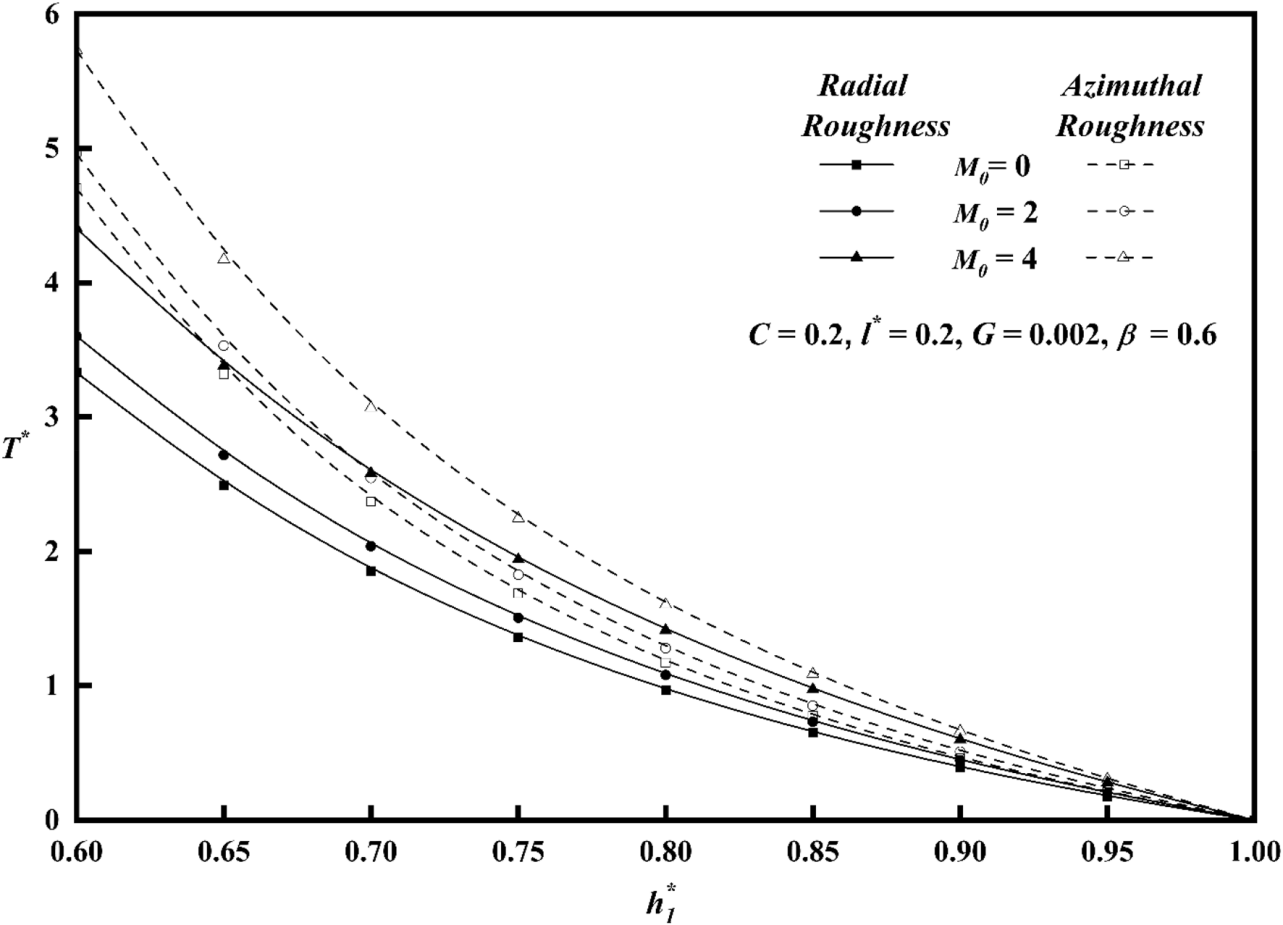
Comparison of $T^{*}$ with $h_{1}^{*}$ on a graph for various values of $M_{0}$.

**Figure 16:**
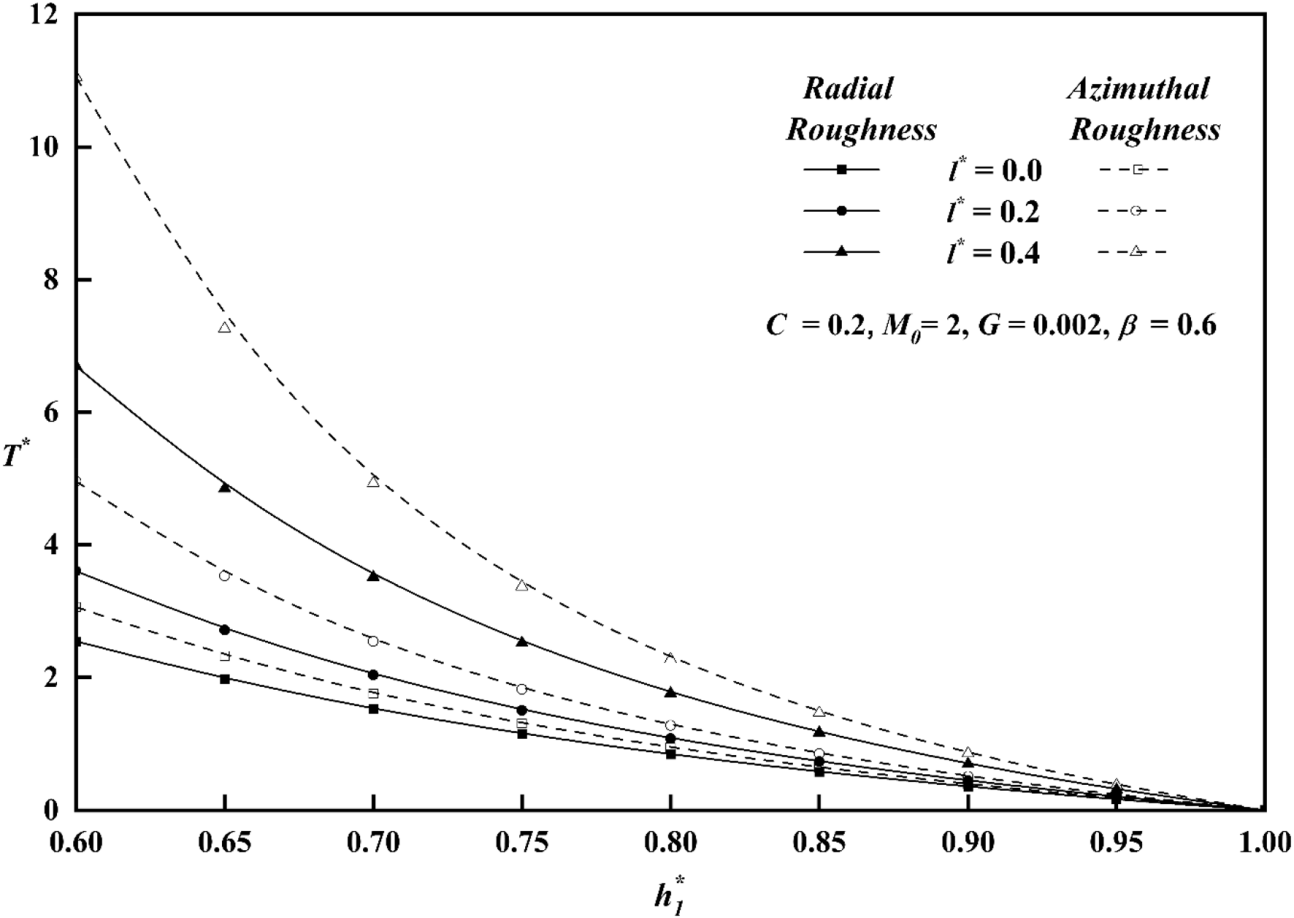
Comparison of $T^{*}$ with $h_{1}^{*}$ on a graph for various values of $l^{*}$.

**Figure 17:**
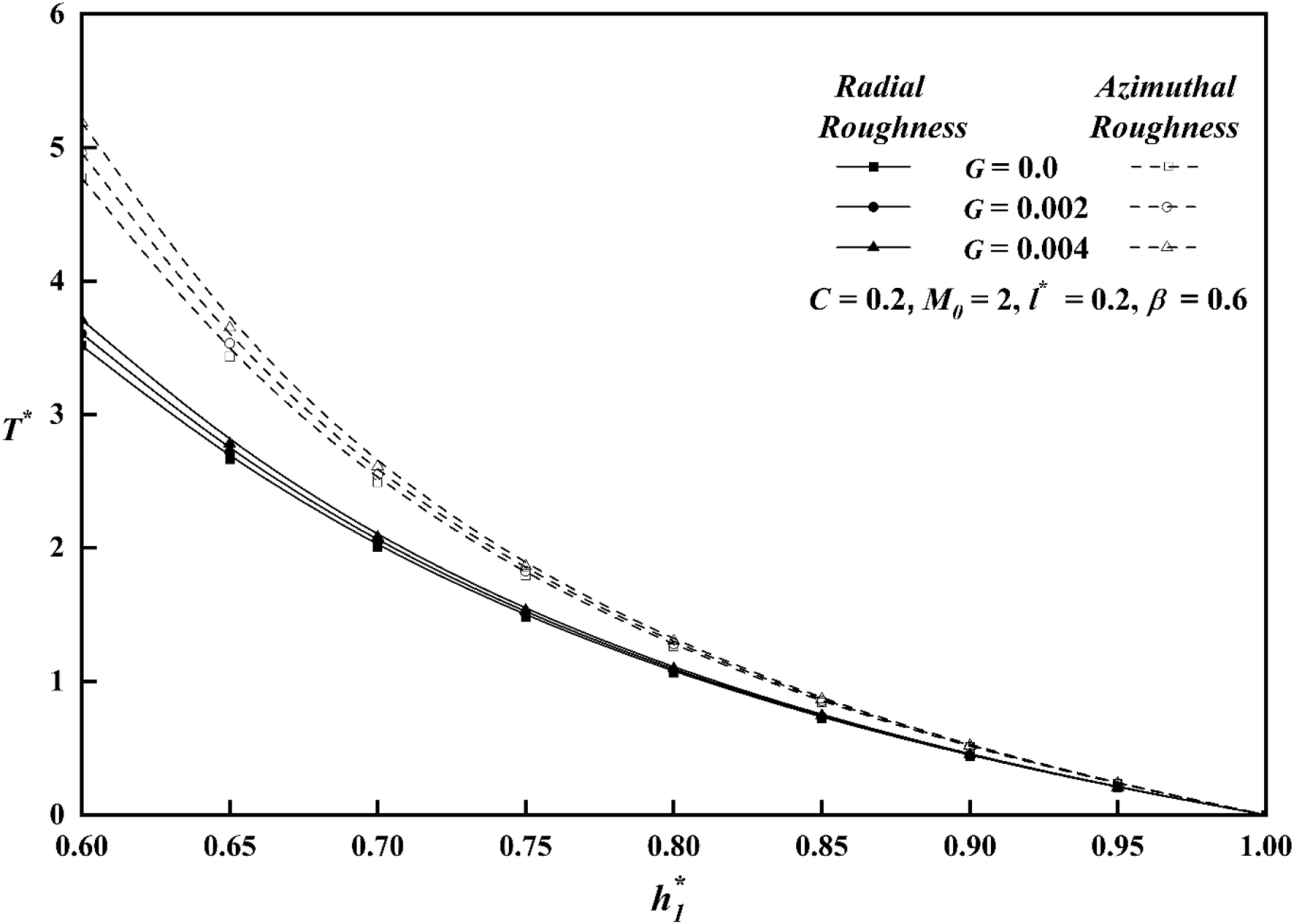
Comparison of $T^{*}$ with $h_{1}^{*}$ on a graph for various values of *G.*

The squeeze film time, or the amount of time it takes for the upper plate to reach a film thickness of $h_{1}^{*}$, is another essential bearing characteristic. For both roughness patterns, Fig. 14 shows the variation of $T^{*}$ along $h_{1}^{*}$ for distinct values of roughness parameter *C*. In contrast to the smooth case (*C* = 0)*,* it is seen that the squeeze film time is longer for the azimuthal roughness pattern, while the radial roughness pattern has a reverse effect, causing $T^{*}$ to decrease. Figure 15 depicts the deviation of $T^{*}$ along $h_{1}^{*}$ for distinct $M_{0}$ values for both rough configurations. The increase in the magnetic parameter is positively correlated with the rise in the squeeze film time for azimuthal pattern when compared to non-magnetic effect $\left( {M_{0} \, = \, 0 \, } \right)$. The presence of an applied magnetic field creates a significant resistance to the flow of fluid in the film region, leading to a substantial accumulation of fluid within this region. As a result, the squeezing time lengthens noticeably, revealing that the magnetic field offers the upper plate's delayed squeeze film, which lowers the coefficient of friction. The graph of $T^{*}$ along $h_{1}^{*}$ as a function of couple stress parameter l^*^ is presented in Fig. 16. The effect of couple-stress parameter is to increase the squeeze time compared to the Newtonian case. The presence of a magnetic field, in conjunction with a couple-stress fluid, enhances the squeeze film of the upper plate, resulting to a decrease in the coefficient of friction and the rate of wear of plates. Figure 17 explains the impact of viscosity parameter $G$, on the variation of $T^{*}$ along $h_{1}^{*}$ for both roughness configurations. Since a fluid with a higher viscosity parameter opposes motion and dissipates energy faster, its squeeze film duration is shorter. It is also observed that as $h_{1}^{*}$ increases, the time $T^{*}$ decreases uniformly across all numerical values.

**Figure 18:**
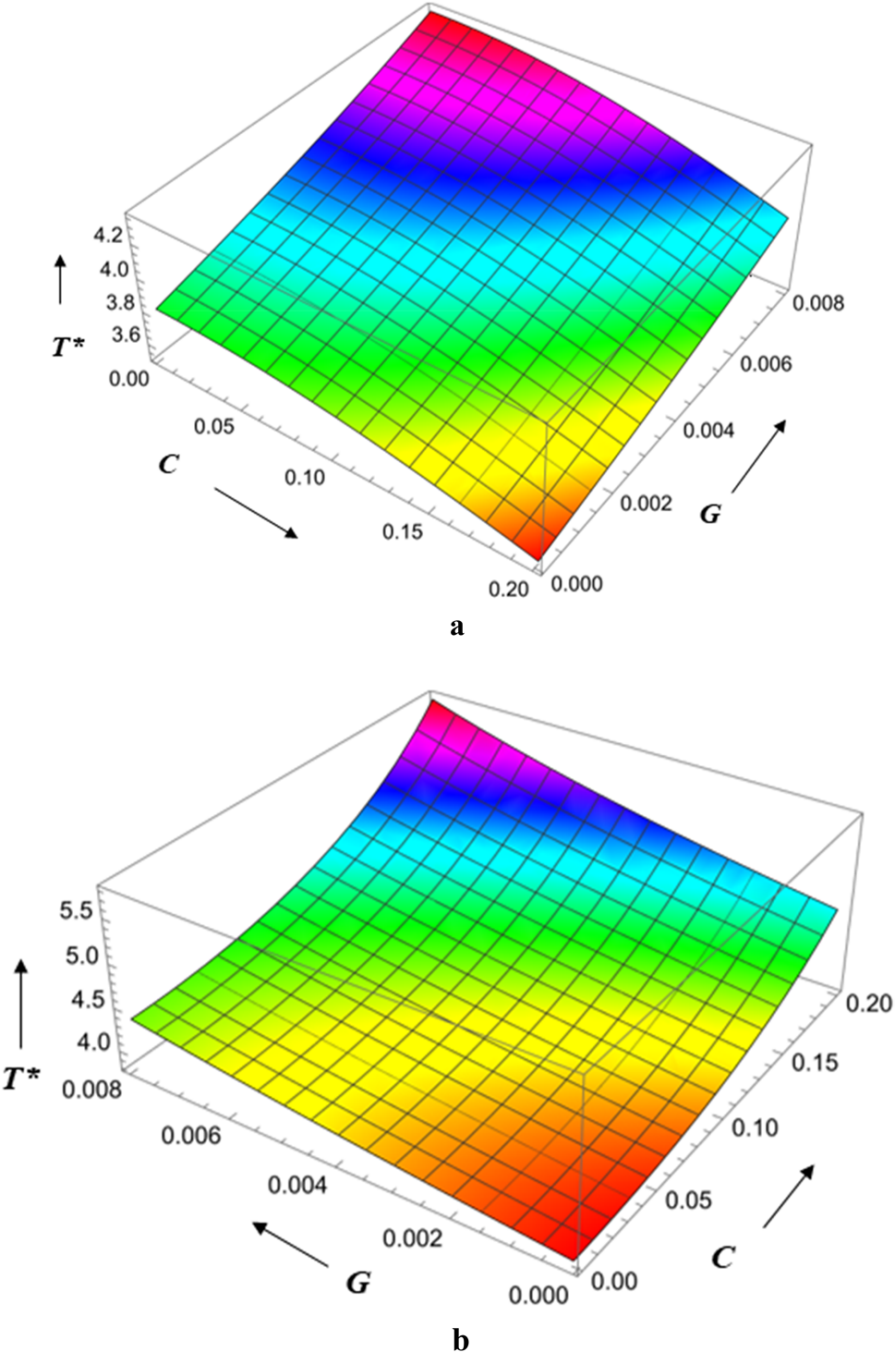
(**a**) Diagram showing how *C* (radial roughness) and *G* influences $T^{*}$. (**b**) Diagram showing how *C* (azimuthal roughness) and *G* influences $T^{*}$.

**Figure 19:**
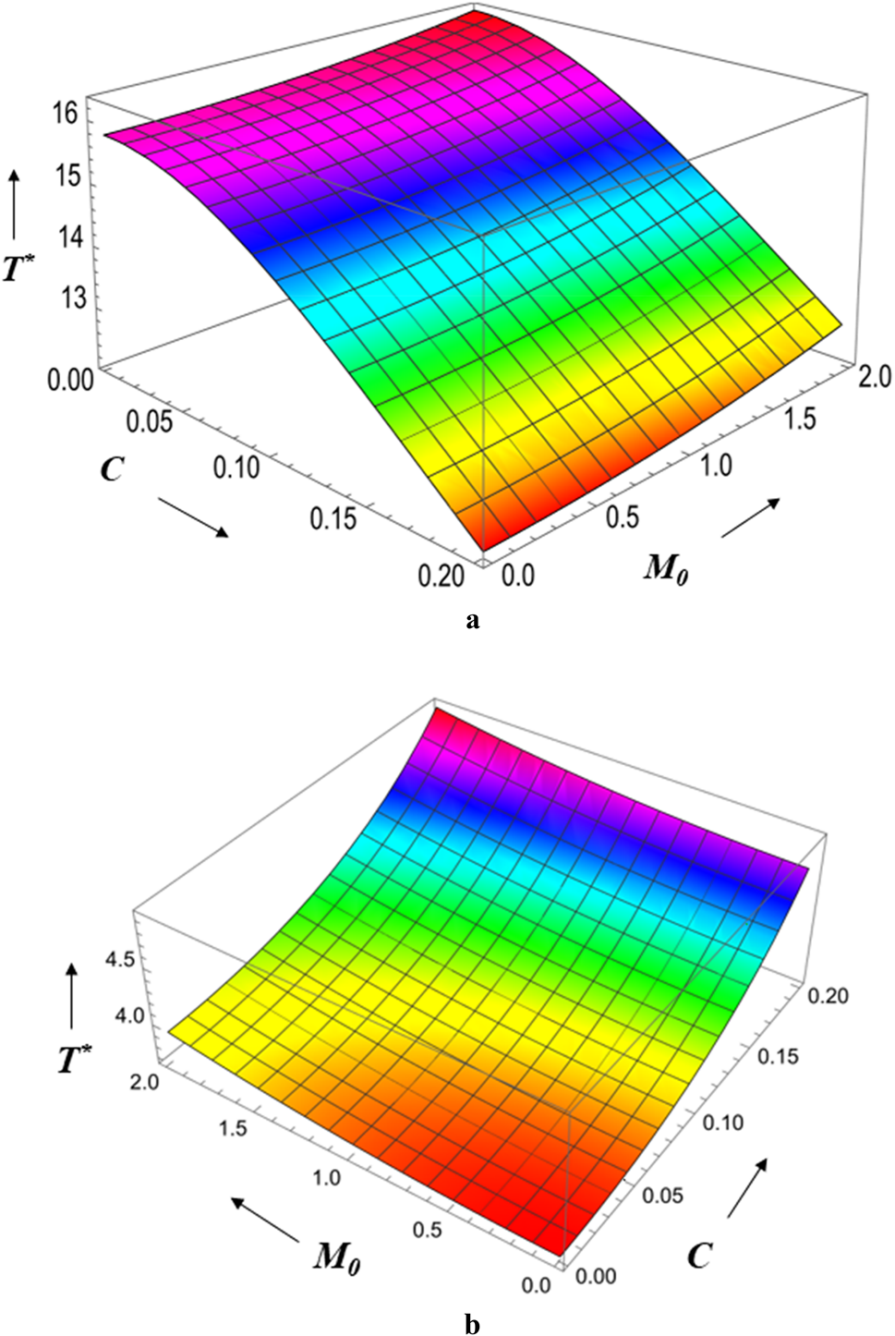
(**a**) Diagram showing how *C* (radial roughness) and $M_{0}$ influences $T^{*}$. (**b**) Diagram showing how *C* (azimuthal roughness) and $M_{0}$ influences $T^{*}$.

Squeeze film time as a function of surface roughness and viscosity variation is shown in Fig. 18a,b. In this scenario, the magnetic parameter is higher, the squeeze film time is delayed, and the radial roughness parameter is at its lowest level. Squeeze film time is lengthened, the magnetic parameter is increased, and the roughness parameter in the azimuthal direction is increased. Squeeze film time is found to be higher for azimuthal roughness than radial roughness as the Hartmann number increases. Figures 19a,b illustrate the influence of the roughness parameter and the magnetic parameter on the squeeze film time. Squeeze film times are shown to be greatest for combinations of high Hartmann number and high azimuthal roughness and shorter for combinations of lower radial roughness parameter and high radial roughness. Squeeze film time is found to be higher for azimuthal roughness than radial roughness as the Hartmann number increases.

#### Frictional force

**Figure 20:**
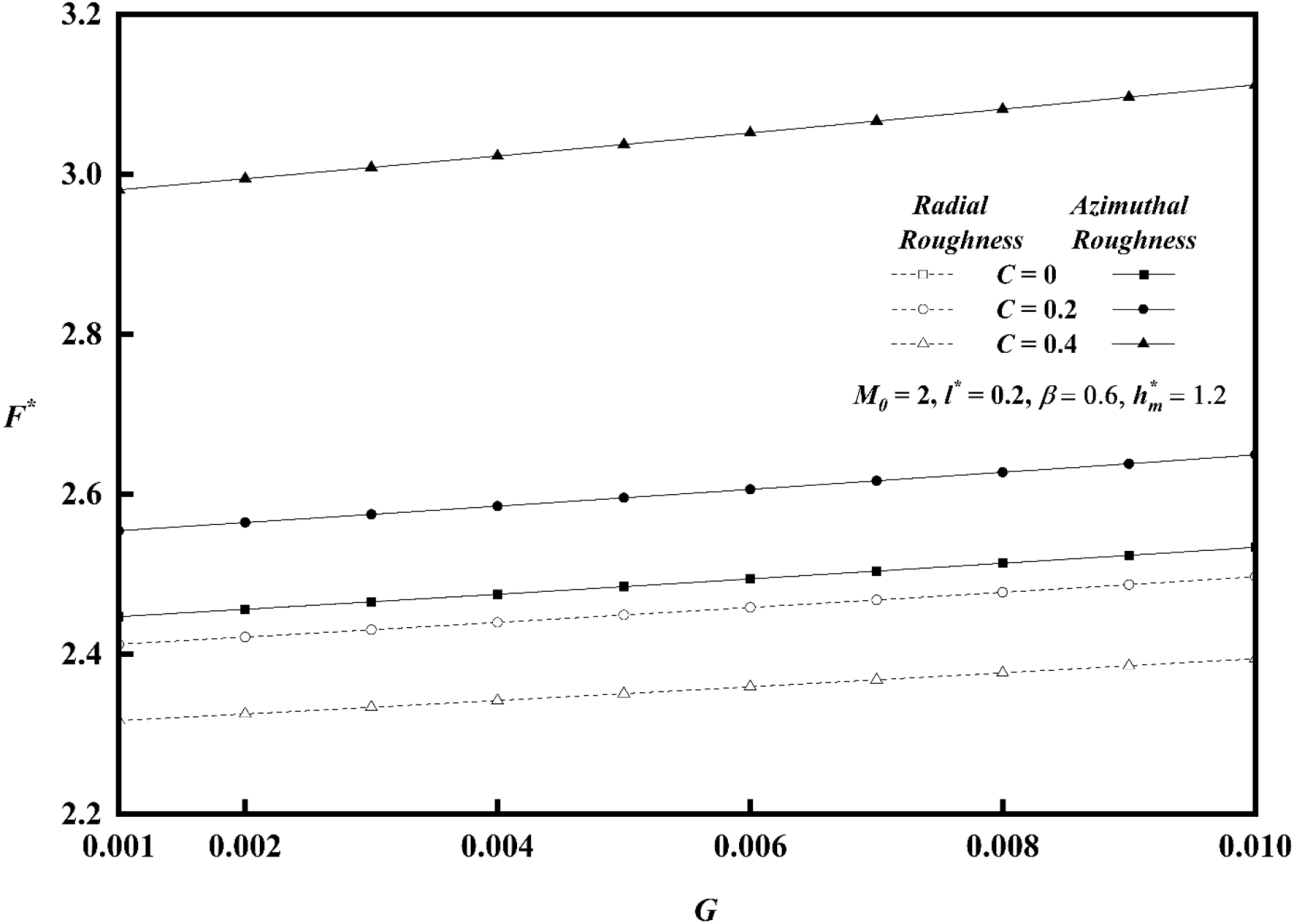
Plot of $F^{*}$ with $G$ for varying values of $C$.

**Figure 21:**
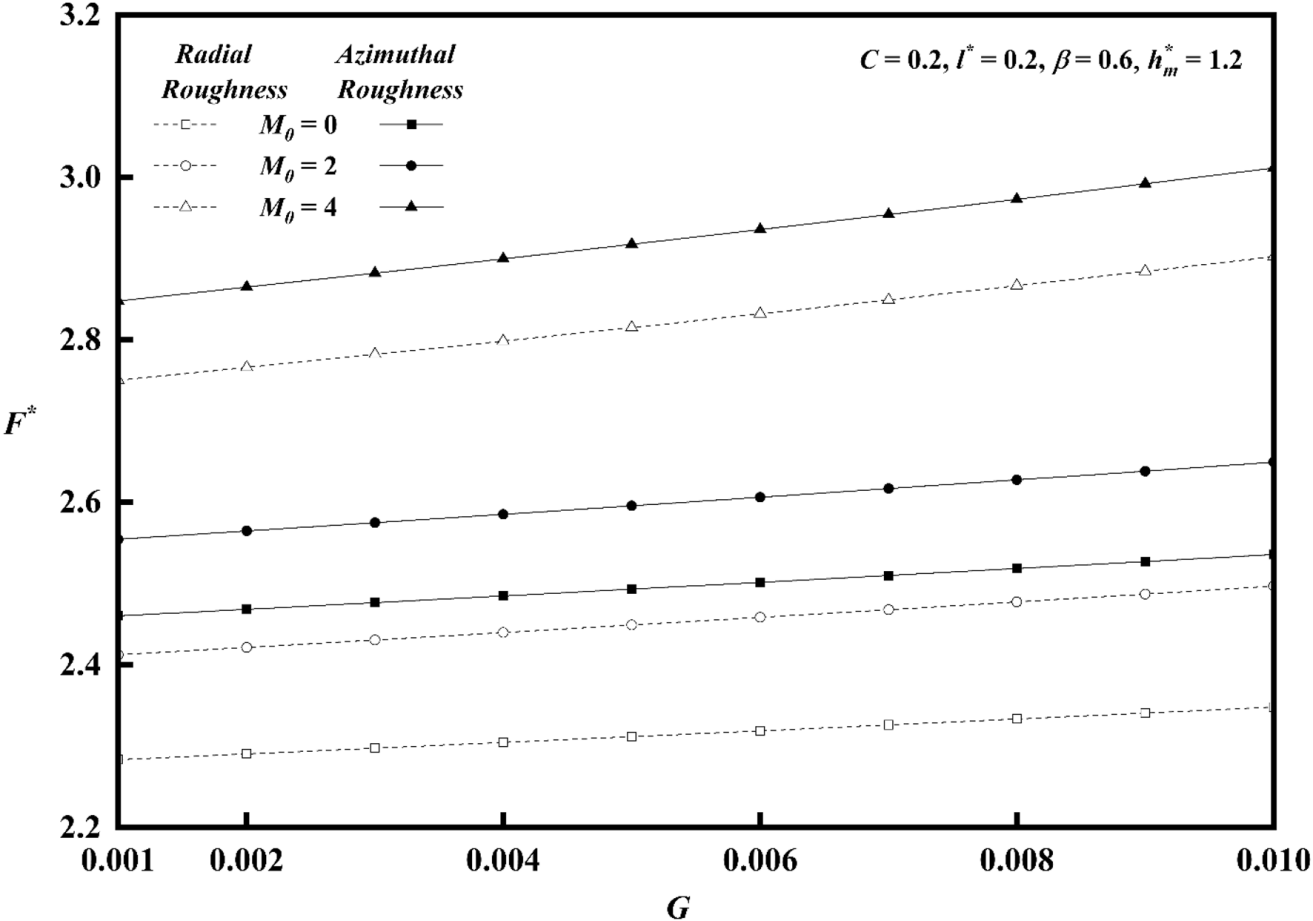
Plot of $F^{*}$ with $G$ for varying values of $M_{0}$.

**Figure 22:**
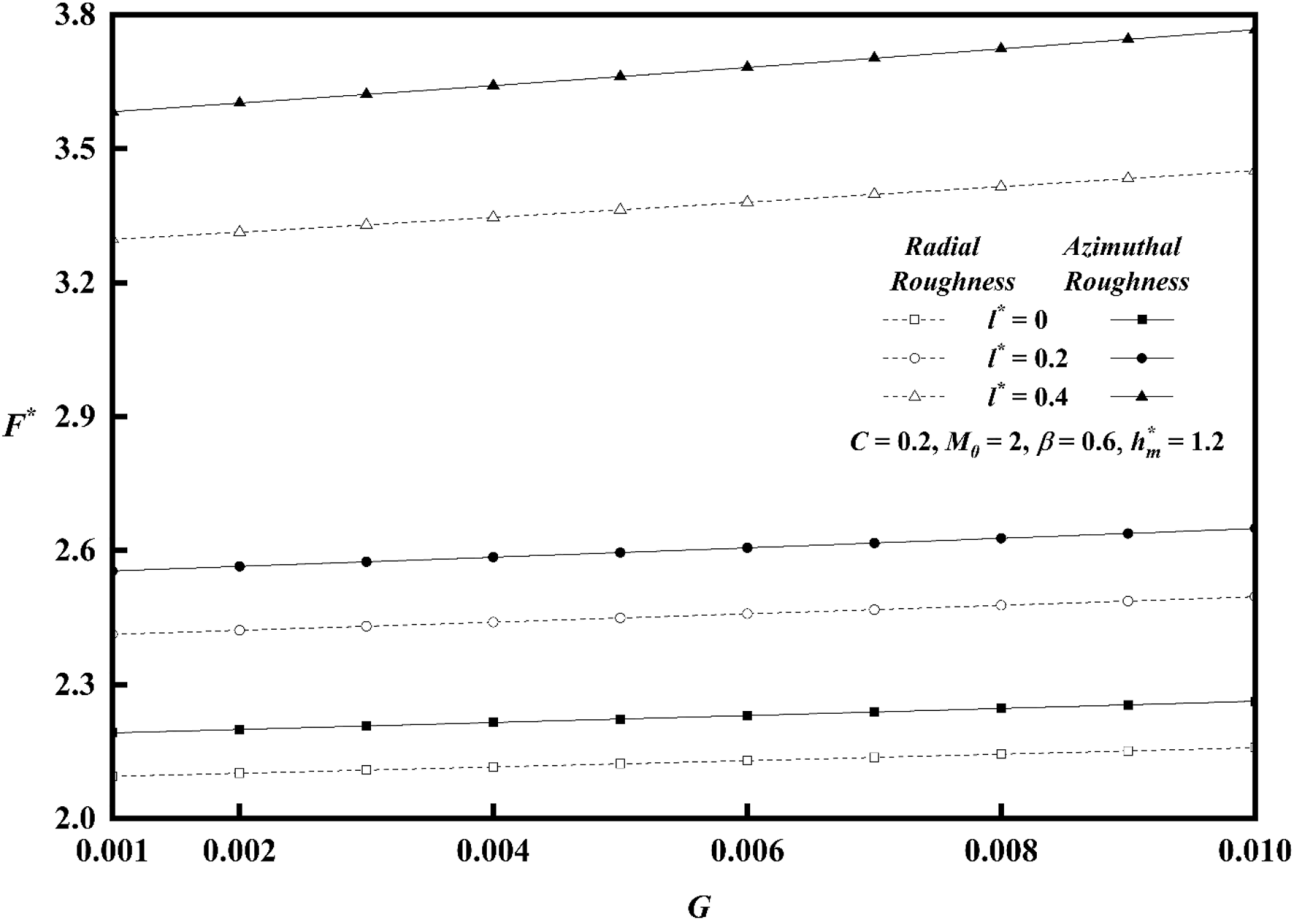
Plot of $F^{*}$ with $G$ for varying values of $l^{*}$.

**Figure 23:**
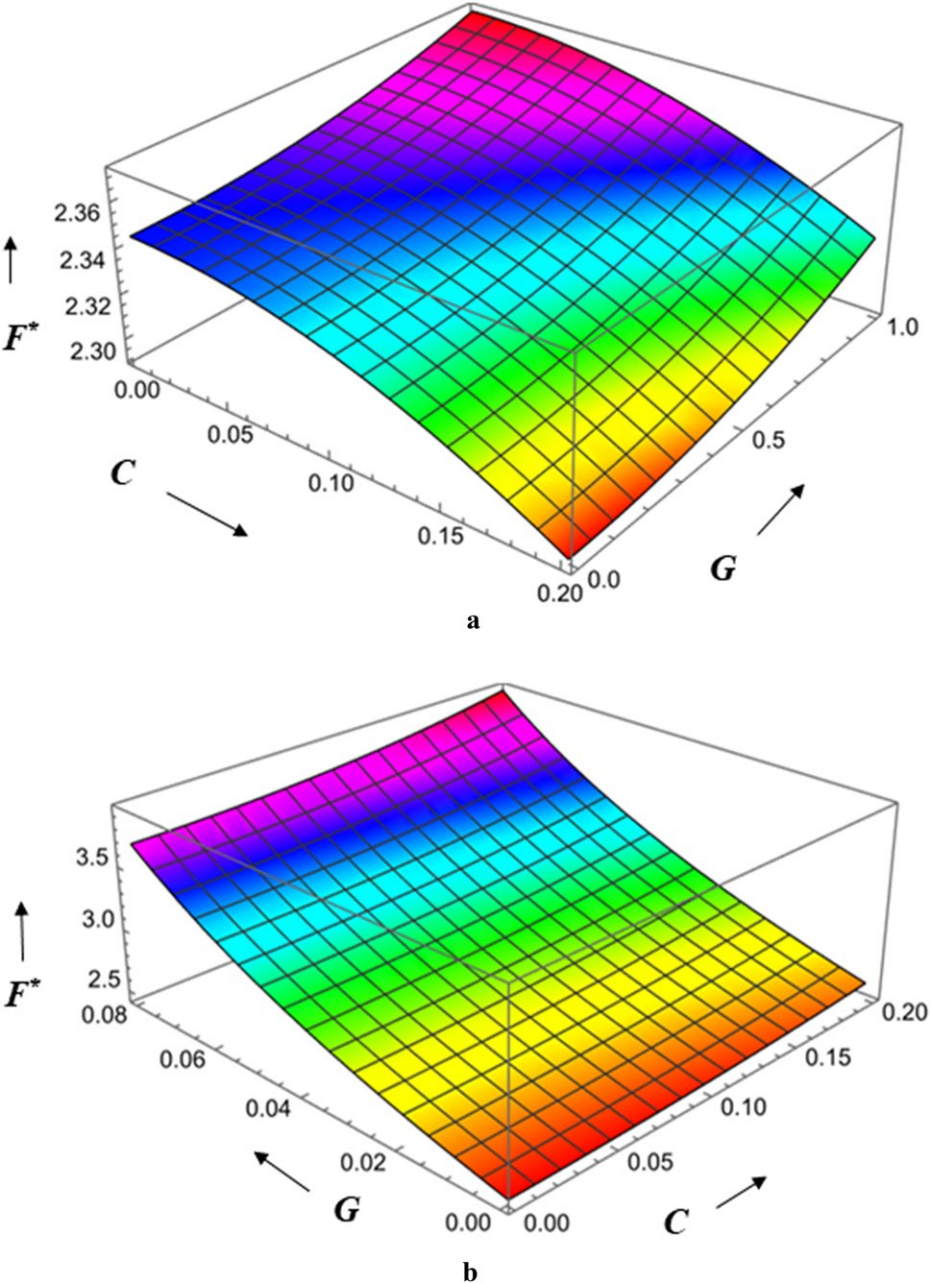
(**a)** Diagram showing how *C* (radial roughness) and $G$ influences $F^{*}$. (**b**) Diagram showing how *C* (azimuthal roughness) and $G$ influences $F^{*}$.

Figures 20, 21, 22 represents variations of frictional force $F^{*}$ versus viscosity parameter *G* as function of roughness parameter, Hartmann number and couple stress parameter for both roughness configurations. As compared to smooth case (*C* = 0), there is increase in $F^{*}$ for larger values of *G.* The impact of couple stress fluid and Hartmann number is significant for azimuthal roughness than radial roughness as compared to Newtonian and Non-Magnetic case. Figure 23a,b show the combined effect of surface roughness and viscosity variation on frictional force $F^{*}$ . For higher values of *G*, there is a decrease in frictional force for radial configuration whereas a reserved trend is noticed azimuthal configuration.

**Figure 24:**
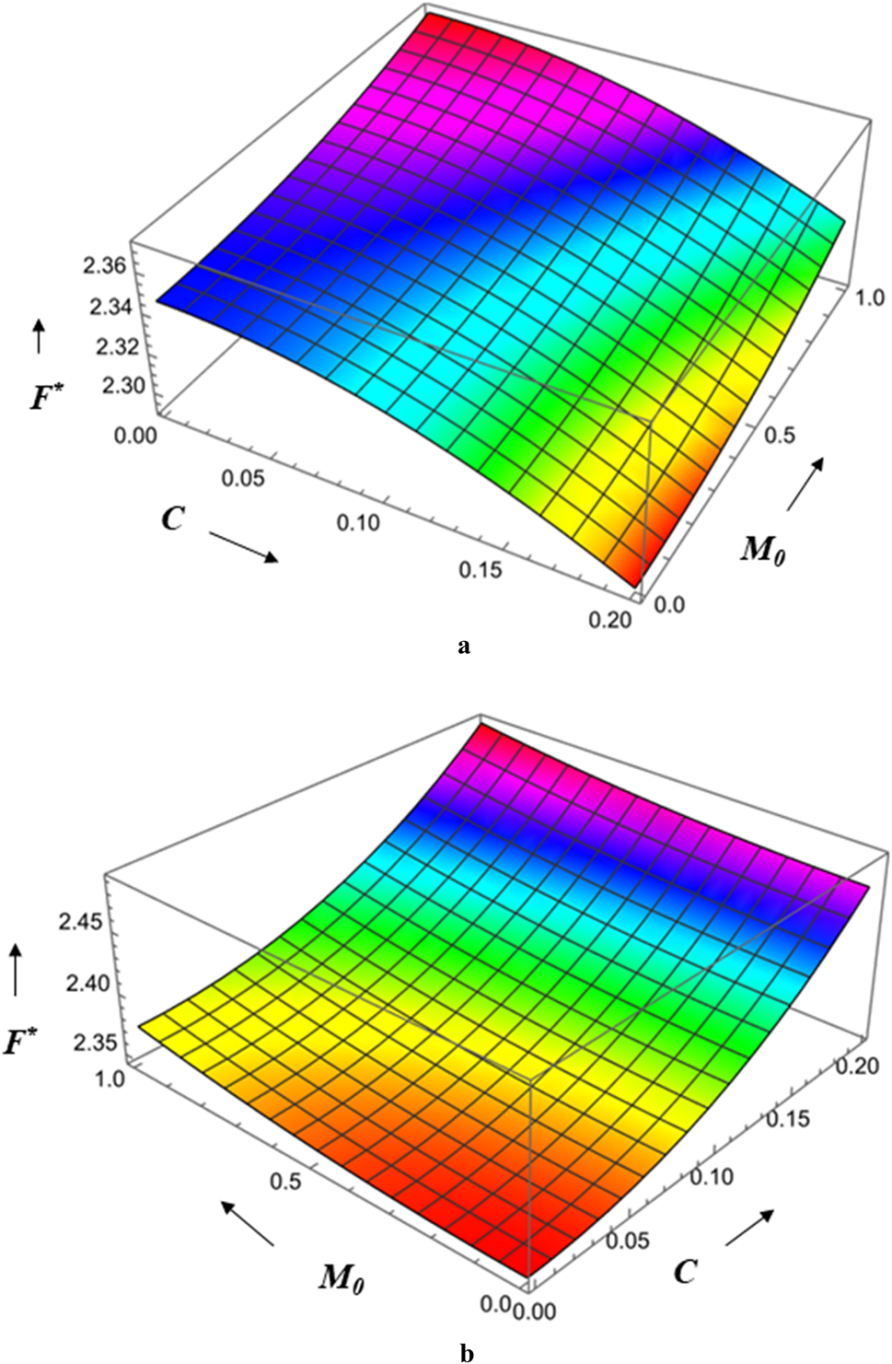
(**a**) Diagram showing how *C* (radial roughness) and $M_{0}$ influences $F^{*}$. (**b**) Diagram showing how *C* (azimuthal roughness) and $M_{0}$ influences $F^{*}$.

Figure 24a,b show the combined effect of the roughness parameter and the magnetic parameter on frictional force $F^{*}$. We observe that the highest friction occurs at small values of the radial roughness parameter and large values of the Hartmann number, and that the same holds true for the azimuthal direction. The Hartmann number has a bigger impact on frictional force for azimuthal roughness than radial roughness.

## Concluding remarks

The influence of surface roughness, MHD and viscosity variation on the couple stress squeeze film characteristics of curved circular and flat plates is analysed based on Christensen^17^ stochastic theory for rough surfaces. According to the results discussed, the following conclusions can be drawn:

- The surface roughness provides a significant influence on the lubrication characteristics (Pressure, load carrying capacity and frictional force) of the bearing. The impact of surface roughness is more pronounced when considering azimuthal roughness patterns compared to radial roughness patterns.
- The consequences of couple stress is apparent when considering an azimuthal roughness pattern in comparison to a radial roughness pattern. The incorporation of additives in the fluid leads to a noticeable enhancement in pressure, load-carrying capacity, squeeze-film time and frictional force compared to the Newtonian case for both types of roughness patterns.
- When compared to the non-magnetic case, the impact of magnetic field, as indicated by the Hartmann number, increases the pressure, load-carrying capacity, squeeze film time and frictional force, for higher values of the Hartmann number.
- In comparison with non-viscos case, increase in viscosity variation parameter, enhances squeeze film characteristics steadily.
- Table 1 illustrates the numerical comparison of present analysis with Lin et al.^53^ and it is observed that in presence of viscosity parameter there is an enchantment in squeeze film characteristics for azimuthal roughness than radial roughness pattern.

**Table 1:** Numerical comparison of present analysis with Lin et al.^53^, with l^*^ = 0.2, *β* = 0.6, *h*_m_^*^ = 0.6, r^*^ = 0.5.

	*M*_0_	Lin et al.^53^	Present analysis			
			*G*→0, *C*→0		*G* = 0.004, *C* = 0.2	
			Radial roughness	Azimuthal roughness	Radial roughness	Azimuthal roughness
P*	0	27.3476	7.3477	27.3476	26.0964	36.0829
	1	27.6455	27.6455	27.6454	26.4755	36.4332
	2	28.5378	28.5379	28.5378	27.6078	37.4858
	3	30.0243	30.0244	30.0243	29.4854	39.2491
	4	32.1047	32.1048	32.1047	32.1018	41.7378
W*	0	47.9578	47.9581	47.9578	48.1683	146.116
	1	48.151	48.1513	48.151	48.5656	147.063
	2	48.7299	48.7302	48.7299	49.7607	149.974
	3	49.6943	49.6947	49.6943	51.7692	155.104
	4	51.0443	51.0446	51.0443	54.6222	162.987
T*	0	7.03865	7.03867	7.03866	6.79573	12.5178
	1	7.09783	7.09786	7.09785	6.87946	12.6095
	2	7.27512	7.27514	7.27513	7.13007	12.8865
	3	7.57045	7.57048	7.57046	7.54744	13.355
	4	7.98371	7.98374	7.98373	8.13274	14.0265

These results are anticipated to assist design engineers in selecting appropriate roughness parameter for a particular magnetic field and lubricant to prolong the bearing life.

## Data Availability

All data used in this manuscript have been presented within the article.

## Appendix

$$\lambda_{11} = \frac{{\lambda_{1}^{2} }}{{(\lambda_{1}^{2} - \lambda_{2}^{2} )}}\frac{{\cosh \left\{ {{{\lambda_{2} (2z - h)} \mathord{\left/ {\vphantom {{\lambda_{2} (2z - h)} {2l}}} \right. \kern-0pt} {2l}}} \right\}}}{{\cosh {{(\lambda_{2} h} \mathord{\left/ {\vphantom {{(\lambda_{2} h} {2l}}} \right. \kern-0pt} {2l}})}}\,\,\,\,\lambda_{12} = \frac{{\lambda_{2}^{2} }}{{(\lambda_{1}^{2} - \lambda_{2}^{2} )}}\frac{{\cosh \left\{ {{{\lambda_{1} (2z - h)} \mathord{\left/ {\vphantom {{\lambda_{1} (2z - h)} {2l}}} \right. \kern-0pt} {2l}}} \right\}}}{{\cosh {{(\lambda_{1} h} \mathord{\left/ {\vphantom {{(\lambda_{1} h} {2l}}} \right. \kern-0pt} {2l}})}}$$

$$\lambda_{1} = \sqrt {\frac{{1 + \sqrt {\left( {1 - {{4M_{0}^{2} l^{2} } \mathord{\left/ {\vphantom {{4M_{0}^{2} l^{2} } {h_{0}^{2} }}} \right. \kern-0pt} {h_{0}^{2} }}} \right)} }}{2}} \,\,\,\,\lambda_{2} = \sqrt {\frac{{1 - \sqrt {\left( {1 - {{4M_{0}^{2} l^{2} } \mathord{\left/ {\vphantom {{4M_{0}^{2} l^{2} } {h_{0}^{2} }}} \right. \kern-0pt} {h_{0}^{2} }}} \right)} }}{2}}$$

$$\begin{aligned} & \lambda_{21} = \frac{{2\cosh \left\{ {{{\left( {z - h} \right)} \mathord{\left/ {\vphantom {{\left( {z - h} \right)} {\sqrt 2 l}}} \right. \kern-0pt} {\sqrt 2 l}}} \right\} + 2\cosh \left( {{z \mathord{\left/ {\vphantom {z {\sqrt 2 l}}} \right. \kern-0pt} {\sqrt 2 l}}} \right)}}{{2\left\{ {\cosh \left( {{h \mathord{\left/ {\vphantom {h {\sqrt 2 l}}} \right. \kern-0pt} {\sqrt 2 l}}} \right) + 1} \right\}}} \\ & \lambda_{22} = \frac{{\left( {{z \mathord{\left/ {\vphantom {z {\sqrt 2 l}}} \right. \kern-0pt} {\sqrt 2 l}}} \right)\sinh \left\{ {{{\left( {z - h} \right)} \mathord{\left/ {\vphantom {{\left( {z - h} \right)} {\sqrt 2 l}}} \right. \kern-0pt} {\sqrt 2 l}}} \right\} + \left\{ {{{\left( {z - h} \right)} \mathord{\left/ {\vphantom {{\left( {z - h} \right)} {\sqrt 2 l}}} \right. \kern-0pt} {\sqrt 2 l}}} \right\}\sinh \left( {{z \mathord{\left/ {\vphantom {z {\sqrt 2 l}}} \right. \kern-0pt} {\sqrt 2 l}}} \right)}}{{2\left\{ {\cosh \left( {{h \mathord{\left/ {\vphantom {h {\sqrt 2 l}}} \right. \kern-0pt} {\sqrt 2 l}}} \right) + 1} \right\}}} \\ \end{aligned}$$

$$\lambda_{31} = \frac{{\cos \left( {B_{2} z} \right)\cosh \left\{ {A_{2} \left( {z - h} \right)} \right\} + \cosh \left( {A_{2} z} \right)\cos \left\{ {B_{2} \left( {z - h} \right)} \right\}}}{{\cosh \left( {A_{2} h} \right) + \cos \left( {B_{2} h} \right)}}$$

$$\lambda_{32} = \frac{{\cot \theta {\kern 1pt} {\kern 1pt} {\kern 1pt} \left\langle {\sinh \left( {A_{2} z} \right)\sin \left\{ {B_{2} \left( {z - h} \right)} \right\} + \sin \left( {B_{2} z} \right)\sinh \left\{ {A_{2} \left( {z - h} \right)} \right\}} \right\rangle }}{{\cosh \left( {A_{2} h} \right) + \cos \left( {B_{2} h} \right)}}$$

$$A_{2} = \sqrt {\frac{{M_{0} }}{{lh_{0} }}} Cos\left( {\frac{\theta }{2}} \right)\,\,\,{\kern 1pt} {\kern 1pt} B_{2} = \sqrt {\frac{{M_{0} }}{{lh_{0} }}} Sin\left( {\frac{\theta }{2}} \right)\,\,\,\theta = \tan^{ - 1} \left( {\sqrt {4l^{2} M_{0}^{2} /h_{0}^{2} - 1} } \right)$$
